# Elevated concentrations of soil carbon dioxide with partial root-zone drying enhance drought tolerance and agro-physiological characteristics by regulating the expression of genes related to aquaporin and stress response in cucumber plants

**DOI:** 10.1186/s12870-024-05310-2

**Published:** 2024-10-01

**Authors:** Emad A. Abdeldaym, Hassan A. Hassan, Mohamed M. El-Mogy, Mohamed S. Mohamed, Mohamed E. Abuarab, Hanaa S. Omar

**Affiliations:** 1 Department of Vegetable Crops, Faculty of Agriculture, Cairo University, Giza, 12613 Egypt; 2 Department of Arid Land Agriculture, College of Agricultural and Food Science, King Faisal University, Al-Ahsa, 31982 Saudi Arabia; 3 Agricultural Engineering Department, Faculty of Agriculture, Cairo University, PO box 12613, Giza, Egypt; 4 Department of Genetics, Faculty of Agriculture, Cairo University, PO box 12613, Giza, Egypt

**Keywords:** Water shortage, Plant performance, Elevated CO_2_ levels, Gene regulation, Physiological responses, *Cucumis sativus*

## Abstract

Water scarcity and soil carbon dioxide elevation in arid regions are considered the most serious factors affecting crop growth and productivity. This study aimed to investigate the impacts of elevated CO_2_ levels (eCO_2_ at rates of 700 and 1000 ppm) on agro-physiological attributes to induce drought tolerance in cucumbers by activating the expression of genes related to aquaporin and stress response, which improved the yield of cucumber under two levels of irrigation water conditions [75% and 100% crop evapotranspiration (ETc)]. Therefore, two field experiments were conducted in a greenhouse with controlled internal climate conditions, at the Mohamed Naguib sector of the national company for protected agriculture, during the winter seasons of 2021–2022 and 2022–2023. The treatments included eCO_2_ in soil under normal and partial root zoon drying (PRD, 100% ETc Full irrigations, and 75% ETc). All the applied treatments were organized as a randomized complete block design (RCBD) and each treatment was replicated six times. Untreated plants were designed as control treatment (CO_2_ concentration was 400 ppm). The results of this study showed that elevating CO_2_ at 700 and 1000 ppm in soil significantly increased plant growth parameters, photosynthesis measurements, and phytohormones [indole acetic acid (IAA) and gibberellic acid (GA3)], under partial root-zone drying (75% ETc) and full irrigation conditions (100% ETc). Under PRD condition, eCO_2_ at 700 ppm significantly improved plant height (13.68%), number of shoots (19.88%), Leaf greenness index (SPAD value, 16.60%), root length (24.88%), fresh weight (64.77%) and dry weight (61.25%) of cucumber plant, when compared to untreated plants. The pervious treatment also increased photosynthesis rate, stomatal conductance, and intercellular CO_2_ concentration by 50.65%, 15.30% and 12.18%; respectively, compared to the control treatment. Similar findings were observed in nutrient concentration, carbohydrate content, Proline, total antioxidants in the leaf, and nutrients. In contrast, eCO_2_ at 700 ppm in the soil reduced the values of transpiration rate (6.33%) and Abscisic acid (ABA, 34.03%) content in cucumber leaves compared to untreated plants under both water levels. Furthermore, the results revealed that the gene transcript levels of the aquaporin-related genes (*CsPIP1-2* and *CsTIP4*) significantly increased compared with a well-watered condition. The transcript levels of *CsPIP* improved the contribution rate of cell water transportation (intermediated by aquaporin’s genes) and root or leaf hydraulic conductivity. The quantitative real-time PCR expression results revealed the upregulation of *CsAGO1* stress-response genes in plants exposed to 700 ppm CO_2_. In conclusion, elevating CO_2_ at 700 ppm in the soil might be a promising technique to enhance the growth and productivity of cucumber plants in addition to alleviating the adverse effects of drought stresses.

## Introduction

Global climate change and the lack of freshwater resources for the agriculture sector and other sectors are the most important determining factors for agricultural production in recent years. According to FAO, there are about 2.3 billion people living in areas with severe water shortages, representing 10% of the world’s population. In addition, about 72% of the fresh water is used in the agricultural sector, and this amount is increasing to cope with the increase in the global population. However, there is a great need to use freshwater in a more efficient way or use other techniques to reduce the use of freshwater in agriculture without a significant reduction in the production. Furthermore, it has been reported that drought stress causes a reduction in growth and production of cucumber [1, 2]. This reduction in growth and production by drought stress is related to many factors including the decrease in the rate of photosynthesis process and chlorophyll content [3], stomatal conductance, electron transfer efficiency and transpiration rate [4].

Partial root drying irrigation (PRD) is a water conservation irrigation technique [5, 6]. The PRD technology delivers water for irrigation in a time and space-dependent way, exposing different areas of the root system to alternating drying/rewetting cycles [7]. Several studies have demonstrated that PRD has a beneficial influence on tomato growth when compared to traditional deficit irrigation (DI) when the irrigation flow is comparable [8, 9]. PRD has the potential to maintain photosynthetic rate (An) while significantly lowering stomatal conductance (Gs), leading to a greater water productivity (WP) [10]. Additionally, it has been exhibited that PRD has favorable impacts on fruit quality indicators as firmness, total soluble solid concentrations, and total sugars [11]. Similarly, PRD may have a favorable impact on fruit’s mineral levels of nutrients [12, 13].

Globally, cucumber (*Cucumis sativus* L.) is one of the most popular vegetables belongs to *Cucurbitaceous* family and classified as a sensitive crop to the drought stress [14]. According to USAD, 100 g of fresh cucumber contains 0.65 g protein, 3.6 g different carbohydrate, 0.5 g fiber, 2.8 g vitamin C, and many other nutrients. Moreover, it has been reported that cucumber contains anti-cancer compounds [15]. However, drought stress reduces the growth, quality, and production of cucumber plants [16–18]. In Egypt, the most cultivated area of cucumber in the winter (from September to April) is under greenhouse conditions. Under greenhouse conditions, the cucumber crop is frequently in a severe CO_2_ shortage state due the reduced ventilation in the winter, which have negative impacts on the photosynthesis processes causes a reduction in yield [19]. There is a need to elevated the CO_2_ concentration in the root zone under greenhouse conditions to cope with the shortage of CO_2_ [20].

During recent years, there has been a rapid increase in interest in determining how plants react to elevated CO_2_ concentrations [21, 22]. Previous studies found that enriching the atmosphere with CO_2_ can enhance the uptake of carbon and improve crop growth and production [23]. It has been reported that an enriched atmosphere with CO_2_ enhances drought stress, which increases yield [24]. Meanwhile, elevated CO_2_ in soil improved growth performance and the productivity of plants grown under abiotic stress conditions, especially water stress conditions [25]. The effect of elevated root zone CO_2_ on plant growth depends on several factors including mineral content, soil pH, abiotic stress factor, and the phenotypes [26, 27]. Some previous works recorded that elevated root zones with CO_2_ enhanced plant growth [28, 29].

An important agronomic strategy to raise the effectiveness and quality of greenhouse vegetables is CO_2_ fertilization. The buildup of secondary metabolites is encouraged by CO_2_ enrichment, which also affects secondary metabolism and improves tolerance to mild drought stress [20]. By controlling the expression of chloroplast-localized proteins, CO_2_ enrichment enhances photosynthesis, oxidative capacity, and osmotic adjustment ability, as well as reduces photo-damage and toxin accumulation under drought stressors [30]. The photosynthesis, biomass, and grain production of contemporary high-yielding wheat cultivars are also promoted by increases in CO_2_ [31]. By boosting photosynthetic rate and water usage efficiency, CO_2_ enrichment improves the growth rate and yield of plants under drought stress [1, 32]. In order to stimulate root growth and lessen the severity of the stress on the plant, it is also important to encourage carbohydrate synthesis and basipetal transport of carbohydrates from the leaves to the root system [33]. These processes have a significant impact on the morphological structure and physiological function of the roots of plants. In order to promote above-ground development, the root system’s metabolic activity rises [34]. Thus, CO_2_ enrichment may control root development and metabolism to counteract the effects of drought-related stress.

Phytohormones are included in the signal regulation of aquaporin and hydraulic conductivity (Lpr) in plant roots cells under stress, and control expression of plant aquaporin genes [35]. Aquaporin shows vital role in enhancing plant water deficit tolerance by stimulating water transportation through cell membrane. Regulating water transportation is essential for plants to acclimatize to water stress [36]. These finding indicate that the growth and improvement of plant, water uptake purpose, and aquaporin expression are connected with the contents of endogenous phytohormones and genes. Nevertheless, slight evidence is accessible on the connection effects of CO_2_ enrichment and drought stresses on water uptake and transport in cucumber plants, and the communication between phytohormone contents and aquaporin expression of root are indefinite. Therefore, to better understand the role of aquaporins in cucumber plants and the impacts of elevated CO_2_ levels on agro-physiological attributes to induce drought tolerance in cucumbers by activating the expression of genes related to aquaporin and stress response and endogenous phytohormone contents of cucumber plants in hydroponic schemes.

In order to improve crop water production and identify specific traits associated with drought acclimatization of cucumber plants, the investigation will focus on the following topics: whether the effect of elevated CO_2_ concentrations (eCO_2_) may promote deficiency tolerance in cucumbers by activating the aquaporin-responsive gene expression, endogenous ABA level, and the antioxidant system; designing water-deficit yield production trials; agronomic approaches; leaf gas exchange; water productivity; cucumber fruit yield; and quality characteristics; and finally, how to evaluate the use of eCO_2_ and water levels to provide economic and ecological advantages, and establish the theoretical and scientific foundation for lowering applied water under eCO_2_ in greenhouse cucumbers.

## Materials and methods

### Plant material and growth conditions

At Mohamed Naguib Agricultural Site, The National Protected Cultivation, EL-Alameen, Egypt (latitude 30.8225 N, longitude 28.9543E, and mean altitude of 20.93 m above sea level), the field experiment was conducted from October 20 to February 21 during the two successive winter seasons of 2021–2022 and 2022–2023 using cucumber plants (*Cucumis sativus* L.) Hybrid Bright cultivar.

Cocopeat Molds were used as an alternative soil in the experimental area because the soil quality is calcareous and contains large amounts of calcium carbonate. Coco Peat, by itself, offers limited nutrients to plants, unlike regular garden soil. However, as a soil-less growth medium, its spongy texture provides increased moisture retention, improved drainage, and better aeration all of which are needed by plants. The dimensions of each mold were 20 cm in width, 100 cm in length, and 30 cm in thickness. The alternative soil (Cocopeat) was chemically analyzed (Table 1).

**Table 1:** Chemical composition of coco peat molds

pH	EC_e_ (ds m^− 1^)	*N* ^+^ (mg kg^− 1^)	*P* (mg kg^− 1^)	K^+^ (mg kg^− 1^)	Fe^2+^ (mg kg^− 1^)	Cu^2+^ (mg kg^− 1^)	Mn^2+^ (mg kg^− 1^)	Zn^2+^ (mg kg^− 1^)
7.85	0.83	90.0	7.44	362.0	0.878	0.052	0.370	0.10

The chemical analysis of irrigation water is provided in Table 2. Irrigation water was collected from a deep well situated in the experimental region, with a pH of 7.89 and a mean electrical conductivity of 0.8 dS m^− 1^.

**Table 2:** Irrigation water chemical analysis at the experimental location

pH	EC (ds.m^− 1^)	TDS (ppm)	Soluble cations (mg L^− 1^)				Soluble anions (mg L^− 1^)				SAR (%)
			Ca^2+^	Mg^2+^	Na^+^	K^+^	${\mathbf{C}\mathbf{O}}_{3}^{2-}$	${\mathbf{H}\mathbf{C}\mathbf{O}}_{3}^{-}$	Cl^−^	${\mathbf{S}\mathbf{O}}_{4}^{2-}$	
7.89	0.8	512.0	71.4	39.61	83.91	8.21	ND	226.38	131.52	163.78	1.99

ND: Not detected

The seeds of cucumber F1 hybrid were purchased from Vitazad Company of Cairo, Egypt. Furthermore, the hybrid seeds used in the current study corresponds to the Egyptian’s guidelines and legislation. The cucumber hybrid used in this research was Bright F1 (https://vitazad.com/product/bright/*).*

### Environmental conditions

The experiment was carried out in a thoroughly controlled greenhouse, and the locale climate was characterized as an arid environment with a chilly winter and a steamy summer. The following average environmental condition variables were recorded daily throughout both cultivated seasons: air temperature in and out of the greenhouse, air relative humidity in and out, and sun radiation from October to February (Table 3).

**Table 3:** Monthly environmental condition variables in the greenhouse as an average for the two cultivated seasons

Growing Season	Climate parameter	Month					
		October	November	December		January	February
2021–2022	T_in_ (^o^C)	21.93	19.98	13.71		12.29	13.36
	T_out_ (^o^C)	20.58	18.90	12.31		10.54	11.43
	RH_in_ (%)	73.32	77.04	77.12		73.66	74.62
	RH_out_ (%)	78.39	80.19	79.75		73.10	75.98
	Solar radiation (W m^− 2^)	369.50	328.10	274.74		299.13	339.76
2022–2023	T_in_ (^o^C)	22.37	20.38	13.98		12.54	13.63
	T_out_ (^o^C)	20.99	19.28	12.56		10.75	11.66
	RH_in_ (%)	74.79	78.58	78.66		75.13	76.11
	RH_out_ (%)	79.96	81.79	81.35		74.56	77.50
	Solar radiation (W m^− 2^)	376.89	334.66	280.23		305.11	346.56

### Irrigation water requirements

The Food and Agriculture Organization of the United Nations often recommends reference evapotranspiration (ETo), which is calculated using daily environmental condition indicators that are measured under greenhouse circumstances and is based on the Penman-Monteith equation. Allen [37] has used the ETo with success. The meteorological data were entered into the ETo calculator software as input:

1 $${\rm{E}}{{\rm{T}}_{\rm{0}}} = {{0.408\Delta \left( {{R_n} + G} \right) + {\rm{\gamma }}\left( {{{900} \over {{\rm{T}} + 273{{\rm{U}}_2}\left( {{{\rm{e}}_s} - {{\rm{e}}_a}} \right)}}} \right)} \over {\Delta + {\rm{\gamma }}\left( {1 + 0.3{{\rm{U}}_2}} \right)}}$$

where ETo represents the reference evapotranspiration (mm day^− 1^), Rn represents net radiation at the crop surface (MJ m^− 2^ day^− 1^), G represents soil heat flux density (MJ m^− 2^ day^− 1^), T represents the mean daily air temperature at 2 m height (°C), U_2_ represents wind speed at 2 m height (ms^− 1^), and es Andean represents saturated vapor pressure deficit (kPa).

The following equation was used to calculate water requirements and schedule watering for the cucumber under a drip irrigation system [38]:

2 $${IR}_{g}=\left(\frac{{ET}_{O}\times {K}_{C}\times {K}_{r}}{{E}_{i}}\right)-(R+LR$$

where, IR_g_ is the gross irrigation requirements (mm day^− 1^), ET_O_ is the reference evapotranspiration (mm day^− 1^), K_C_ is the crop coefficient (FAO-56), K_r_ represents the ground cover reduction factor and the values of Kr will be measured by Keller Eq. (3) [39] as following:

3 $${K}_{r}=GC+0.15(1-GC)$$

where GC is the ground cover (%) would be determined through dividing the shaded area per plant over the whole plant area, E_i_ is the irrigation efficiency (%), R represents the water received by plant from sources other than irrigation (mm), and LR is the amount of water required for the leaching of salts (mm).

A comparison was made between the irrigation water requirements calculated through the Penman-Monteith equation and the actual water needs of the plant by placing subsurface drainage pipes under the coco peat molds in various locations along the experiment area to determine the amounts of drainage water and adjust the amount of irrigation water applied accordingly to save irrigation water and implement sustainable water management.

### System installation and experimental treatments

The fully climatic controlled greenhouse with a polyethylene cover was used, the greenhouse is 10,500 m^2^, 100 m width, 105 m length and 9 m height, the experimental treatment were applied using randomized complete block design (RCBD), where the blocks were the partial root zone drying (PRD, 100% Full irrigation, and 75%) while the treatments were the CO_2_ concentration with three levels of CO_2_ concentration were applied; normal CO_2_ concentration (CO_2_), 400 ppm), elevated CO_2_ concentration (*e*CO_2_), 700 ppm and 1000 ppm) respectively, to seek the maximum yield, water productivity of cucumber. A field plot of 50 $\times$ 32.4 m was selected for the experimental studies. The field plot was divided into 6 equal plots of 50 $\times$ 5.4 m. Each plot included three rows 80 cm apart, 40 cm between plants and represented a single treatment with three replicates.

Installation of the subsurface trickle irrigation system commenced in September 2021 in a controlled facility, which included a screen filter with backflush mechanisms, and a fertilizer injection system, i.e., the Venturi meter. A subsurface trickle tape (Euro drip GR) was carefully placed straight in the ridges, and the tape strips had openings on their upper sides. The installed trickle system had drippers spaced 40 cm apart, each with an application rate of 2.4 L h^− 1^.

The land was prepared for cultivation and was planted using Cocopeat *Molds* and mulch was placed on it. The seeds were planted in germination trays at the end of September, and they were transported and planted in the greenhouse in mid-October. The first carbon dioxide injection was performed 15 days after planting the seedlings. Then follow the injections with all irrigation events.

Liquefied carbon dioxide cylinders weighing 6 kg were used to pump it with the irrigation water in the lateral lines, with a flow meter used to control the amount of carbon dioxide that is injected into the soil with the irrigation water and to adjust the different injection rates, as mentioned in the CO_2_ concentration treatments used to increase the concentration of carbon dioxide in the soil. A pressure relief valve was also used to adjust the carbon dioxide pressure to match the water pressure in the lateral lines, which is around 1 bar, while the carbon dioxide pressure in the cylinders is around 70 bar. Eighteen ball valves (16 mm) were installed to control the amount of CO_2_ injection with irrigation water through laterals with inline emitters for each plot.

### Measurements

#### Water productivity (WP)

WP is an indicator of effectiveness use of irrigation water for crop production. WP examined crop to be calculated according to the method described previously [40] as follows:

4 $$WP=\frac{{E}_{y}}{{\text{I}}_{r}}$$

where, WP is the water productivity of examined crop (kg m^− 3^), E_y_ is the economical yield (kg ha^− 1^), and I_r_ is the amount of irrigation water applied (m^3^ ha^− 1^).

#### Growth measurements

Ninety days after transplantation, the plant samples were randomly selected for recording growth measurements. These measurements included plant height, Number of leaves, leaf area, plant fresh weight, plant dry weight, number of shoots per plants, leaf greening index (SPAD value) and root length. A meter tape was used to measure the plant height and root length. Digital balance was used to assess the plant fresh and dry weight. Leaf area of the fifth full expanded leaf from the top were determined using leaf area scanner. Leaf greening index was determined using SPAD meter (SPAD 502 Minolta Co, Osaka, Japan). Four SPAD readings were taken around the fifth leaf of cucumber plants and the readings average was calculated.

#### Photosynthesis measurements

Photosynthesis parameters included net photosynthesis rate, leaf stomatal conductance, intercellular CO_2_ concentration, and water use efficiency. These parameters were estimated by LICOR 6400 (Lincoln, NE, USA) at the fifth leaf from the top using six plants per treatment. Photosynthesis parameters were performed at mid-day (12.00–2.00 am).

#### Fruit quantity and quality

The cucumber fruits were harvested 2–3 times per week and the average fruit weight; number of fruits per plant, and total yield were recorded for the two successive growing seasons. The early yield of each treatment was calculated by summation of the first four harvestings. The total soluble solids of cucumber fruit were measured using a digital refractometer (model PR101, Co. Ltd., Tokyo, Japan). Fruit hardness was assessed using a firmness tester. Other fruit qualities such as fruit nutrient content, fruit carbohydrate content, fruit Proline content and total antioxidant of fruits are mentioned below.

#### Macronutrient quantification

The macronutrient content was quantified in dry samples of leaves and fruits of cucumber plants. The fresh samples of leaves and fruits were dried using the air-forced oven at 70 ºC for 3 days. Then, the dried samples were ground into fine powder to determine the N, P, K, Ca, and Mg concentrations.

Total nitrogen content (N) was assessed using the Kjeldahl method with minor modifications, as described by Jackson [41]. Approximately 0.5 g of sample either leaves or fruits was mixed with an acidic solution containing sulfuric and per-chloric acids. Then the mixture was heated at 50 ºC for 10 min until the clear solution. After cooling the solution, total nitrogen content was determined using steam distillation, in the presence of 80 mL of NaOH (40%), and titration with sulfuric acid (0.1 N).

Total phosphorus concentrations (P) were determined in dried samples according to the method stated by Chen et al. [42]. Briefly, a 100 mg of dried fine powder was digested with 5 mL of sulfuric acid (98%) and 3 mL of hydrogen peroxide (30% v: v). After cooling to room temperature, the digested sample was diluted to 100 mL with deionized water. The concentration of P in obtained solution was measured using the molybdate blue with absorbance read at 700 nm wavelength on a spectrophotometer.

The concentration of potassium (K), calcium (Ca) and magnesium (Mg) determined in dried samples using the technique described by Junsomboon1 and Jakmunee [43]. Briefly, a 1 g of ground samples homologized with a mixture containing 5 mL of HCl and 50 mL of water. The homogenate was digested and heated using a hot plate for 15 min. After cooling the digested solution, it was filtered using a filter paper Whatman No. 42. the filtrate was adjusted to 100 mL using a volumetric flask (100 mL) by adding distilled water. the concentrations of were assessed in final solutions using the flame photometer apparatus.

#### Total carbohydrates quantification

Total carbohydrates in either leaves or fruits were assessed using the phosphomolybdic acid method [44]. Approximately 2 g of samples were homogenized with 10 mL of 80% ethanol. The mixture was filtered through the Whatman filter paper (No. 1). The collected residues were transferred into a conical flask (250 mL) then 150 mL of distilled water and 5 mL of concentrated HCL (95%). The residue was hydrolyzed for 30 min and cooled to room temperature. The Na_2_CO_3_ was added slowly until the extract became neutral (pH = 7). The filtrate was transferred into a conical flask and condensed in a water bath for 4 min. Then, 0.5 mL of filtrate sample was transferred into a glass tube and 1 mL of reagent (Somogy’s) was added. Then the obtained aliquot was diluted and measured spectrophotometrically at wavelength 560 nm. The findings of total carbohydrates were reported as a percentage (%).

#### Total antioxidant activity

Total antioxidant activity, is indicated to the total antioxidant compounds, was quantified using the technique defined by Zhang et al. [45]. Briefly, 4 g of sample was homogenized in 40 mL of methanol solvent (ethanol: 0.1 MHCl—85:15%, v/v) and sonicated for 10 min. the homogenate was filtrated and the extract was collected. For this assay, 0.2 mL of aforementioned extract was liquefied in 3.8 mL of a methanol DPPH solution. The obtained mixture was gently shaken and preserved at ambient temperature for 30 min in the darkness. The absorbance was measured at wavelength 517 nm. The antioxidant activity was defined as a percent of inhibition according to following Eq. (5):

5 $$\begin{aligned} &{\text{Antioxidant}}\,{\text{activity}}\left( \% \right) \\ & \quad = \frac{{{A_{517\,nm}}\,{\text{of}}\,{\text{DPPH}}\,{\text{solution}} - {A_{517\,nm}}\,{\text{of}}\,{\text{sample}}}}{{{A_{517\,nm}}\,{\text{of}}\,{\text{DPPH}}\,{\text{solution}}}} \times 100 \\ \end{aligned}$$

#### Free proline quantification

The free Proline was extracted from either leaf or fruit tissues of cucumber plants according to the procedure stated by Bates et al. [46]. Approximately 50 mg of sample homogenized with 1 mL of ethanol: water (60:80 v/v). The obtained homogenate was left overnight at 5 ^º^C and then centrifuged at 15,000 rpm for 4 min. A total of 1 mL of extract was diluted with 10 mL of distilled water. Then, a mixture containing 5 mL of ninhydrin and 5 mL of glacial acetic acid was added, and placed in a boiling water bath for 60 min at 100 ºC. The reaction was arrested by placing the test tubes in cold water and the chromophore was extracted with 4 mL toluene. The pooled supernatants were measured spectrophotometrically at wavelength 520 nm.

#### Plant hormones bioassay

Determination of phytohormones such as IAA, GA3 and ABA was reported according to the method stated by Vogel [47]. Briefly, a 10 mg of freeze-dried cucumber leaves were ground into a fine powder. The ground samples were washed with a mixture containing 80% methanol and 2,6-bis (1,1-dimethyl ethyl)-4-methylphenol in dark condition at 5 ºC. The obtained extract was centrifuged (4000 rpm), dehydrated, filtered and evaporated under vacuum at 35 ºC. The concentration of the IAA, GA3 and ABA was determined using Ati-Unicum gas liquid chromatography.

#### Plant defense gene expression and the transcript levels of aquaporin and drought-related genes

To gain an in-depth understanding of the regulation and molecular mechanism of leaf gas by the interaction between PRD and CO_2_ during greenhouse cucumber precipitation. The two aquaporin-related genes and stress-responsive genes, i.e., TIP (CsTIP1-1) and PIP (CsPIP1-2), and Argonaut (CsAGO1), respectively, were selected, and their transcript analysis was achieved using quantitative real-time PCR. These genes have been exposed in cucumber plants in response to the negative effects of drought and e(CO_2_) on leaf quality.

RNA was isolated from leaf plants collected during sixty days of water deficit stress. 100 mg of cucumber plants were extracted using the RNeasy^®^ Plant Kit (Qiagen, Germany). The RNA concentrations were measured using a Nano drop ND-100 spectrophotometer. The Prime-Script First Stand cDNA Synthesis Kit was used to convert RNA to cDNA (thermo kit). The cDNA synthesis reactions were incubated at 37 degrees Celsius for 15 min and at 85 ºC for 5 s. The cDNA achieved was charity in q RT-PCR experiments. Primers specific to one stress-responsive and two aquaporin-related genes were recycled for PCR amplification. Primer Blast (https://www.ncbi.nlm.nih.gov/tools/primer-blast/) or primer 3 (https://primer3.ut.ee/) software was designed to design primers specific to aquaporin and drought-expressed related genes as shown in (Table 4). The primers were used to identify bands of 100–250 bp in length. The Thermal Cycler Bio-Rad Real-Time System II (TaKaRa, Shiga, Japan) and the SYBR kit were functional for real-time PCR. In 96-well plates, the QRT-PCR analysis was done in triplicate. For a total volume of 25 µL, 12.5 µL of SYBR, 1 µL of 60 ng cDNA, 5 µL of 2 mol L^− 1^ primers, and 6.5 µL of DNase-free nuclease water were added. The real-time system’s thermal profile was encompassed by one step at 95 °C for 30 s, then 40 cycles at 95 °C for 5 s, and 60 °C for 30 s. As an internal standard, the actin gene was employed (the housekeeping gene).

### Statistical analysis

The collected data were subjected to testing normality (Shapiro-Wilk test) and homogeneity of variances (Bartlett’s test) of the residuals prior to ANOVA [48, 49]. The combined data from the two seasons were subjected to ANOVA using the IBM SPSS Statistical software program (version 25), followed by Duncan multiple range tests (*P* ≤ 0.05) to determine significant differences between treatments. An online statistical analysis and visualization software performed an analysis of Pearson’s analysis and Heatmap correlation [50].

## Results

### Irrigation water applied and water productivity

The water productivity under full irrigation (100% ETc) was higher than drought stress conditions (75% ETc). In the same context, the highest water productivity was observed under full irrigation comparing with drought stress conditions, where the highest water productivity was 59.22 kg m^− 3^ for 100% ETc with 700 ppm CO_2_ concentration followed by 75% ETc with 700 ppm CO_2_ concentration with 55.14 kg m^− 3^, while the lowest water productivity was 30.31 kg m^− 3^ under 75% ETc with 400 ppm CO_2_ concentration (Table 4). The water productivity has a direct relationship with CO_2_ concentration until 700 ppm then it gets down at 1000 ppm but with higher values compared with reference CO_2_ concentration at 400 ppm. There was not a significant difference between water productivity under 75% ETc with 700 ppm CO_2_ concentration and 100% ETc with 1000 ppm CO_2_ concentration which confirm the availability to save water without decreasing yield and achieving high water productivity, where it ranked at the second order after 75% ETc with 700 ppm CO_2_ concentration with reduction by 7.4%.

**Table 4:** Primers nucleotide sequence charity for quantitative real time -PCR analysis

Serial number	Gene name	Primer name (forward/reverse)	Nucleotide sequence (5 to 3)
1	CsTIP4	CsTIP4-1 F CsTIP4-1R	5-CCCGAAGAGCTCACCAAAT-3 3-CTTCCGTGGGAGTAACCAATAA-5
2	CsPIP1-2	CsPIP1-2 F CsPIP1-2R	5-TTGGGAGCGGCCATTATTTA-3 3-TGAAAGGGATGGCTCTGATTAC-5
3	CsAGO1	CsAGO1-F CsAGO1-R	5-ACACCGTGGAAATTGTTAGGC-3
			3-ACTTGAAGGCAAGGGAGATG-5
4	*CsActin*	Cs*Actin*-F	5-CAACCACAAGGGCTAACAGAG-3
		Cs*Actin*-R	3-GAATCCAGCACGATACCAGT-5

### Vegetative growth parameters of cucumber plants

#### Plant height

**Fig. 1:**
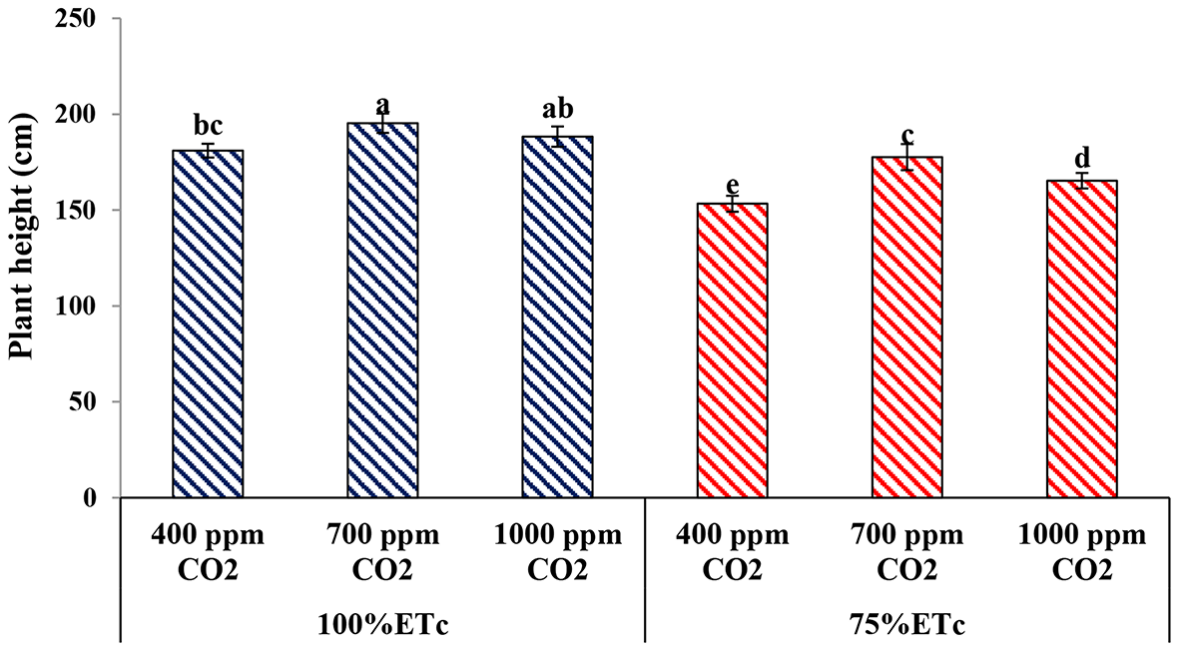
Effect of interaction between water levels and elevated CO_2_ levels on plant height of cucumber plants grown under greenhouse condition. Different letters indicate significant differences between treatments (Duncan’s multiple range test at *P* < 0.05)

As presented in Fig. 1, plant height was significantly affected by PRD and eCO_2_ (P). The plant height of cucumber plants decreased under PRD conditions compared to full irrigation conditions. Under both water regimes, the maximum values of plant height were observed in cucumber plants grown exposed to 700 ppm of CO_2_ followed by the plants treated with 1000 ppm of CO_2_ compared to untreated plants (400 ppm CO_2_).

#### Number of leaves

**Fig. 2:**
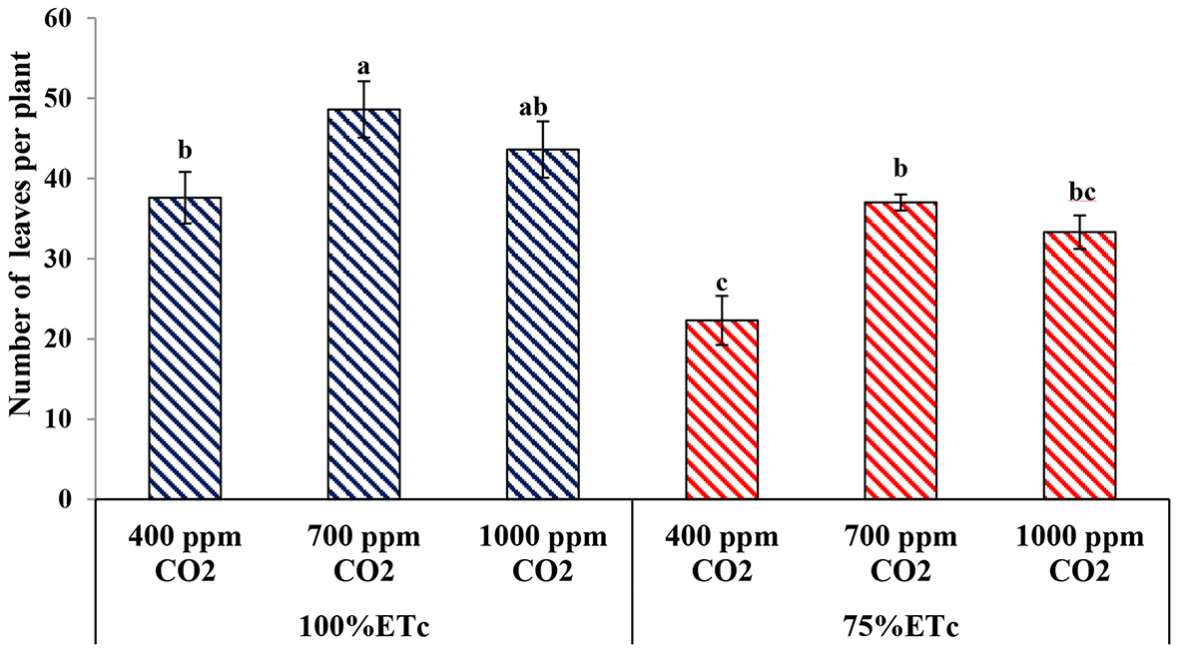
Effect of Effect of interaction between water levels and elevated CO2 levels on number of leaves per plant. Different letters indicate significant differences between treatments (Duncan’s multiple range test at *P* < 0.05)

The cucumber plants exposed to PRD (75 ETc) showed a significant reduction in the number of leaves per plant in comparison to the full irrigated plants (*p* ≤ 0.05). Under both water regimes, the application of 700 ppm CO_2_ significantly enhanced the number of leaves compared to the control, while there was no significant difference between both CO_2_ concentrations. Additionally, there was no significant difference in the number of leaves between the control treatment (under a full irrigation condition) and the 700 ppm CO_2_ treatment (under a PRD condition, Fig. 2).

#### Leaf greenness index (SPAD value)

**Fig. 3:**
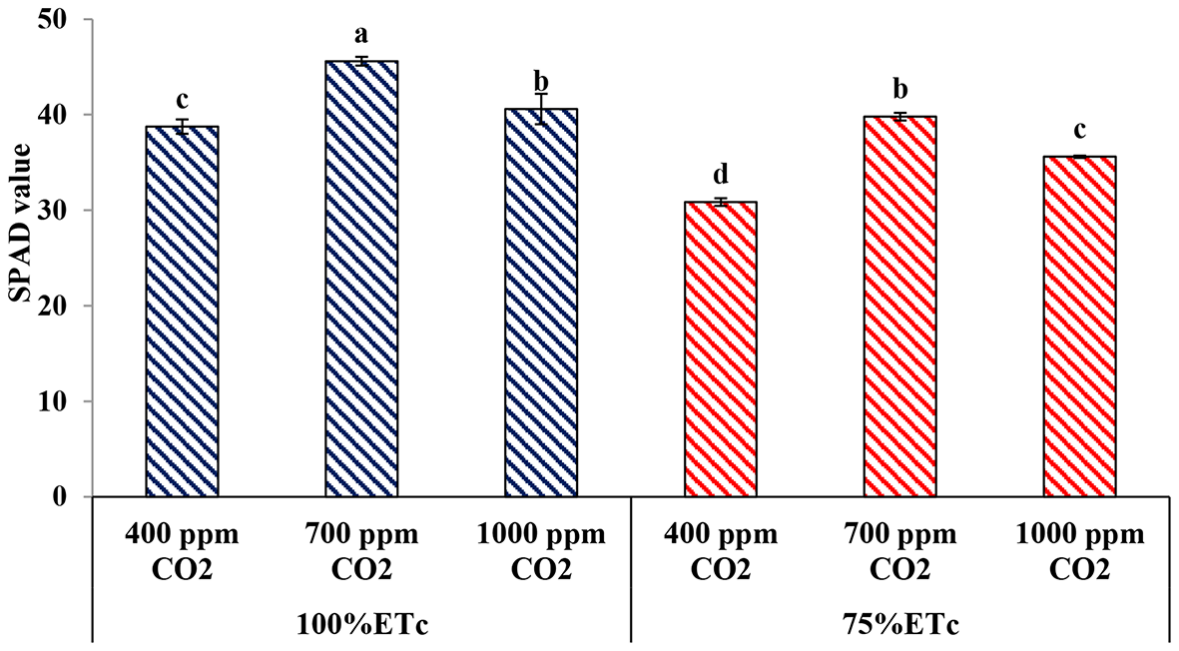
Effect of interaction between water levels and elevated CO_2_ levels on SPAD reading. Different letters indicate significant differences between treatments (Duncan’s multiple range test at *P* < 0.05)

Under PRD conditions, the leaves of plants had a lower SPAD reading compared to the plants that received the recommended water level. Under full irrigation conditions, the plants that received 700 ppm CO_2_ had the highest SPAD values (45.6), followed by 1000 ppm CO_2_ (40.6), while the control plants had the lowest SPAD values under both water levels. Moreover, the SPAD reading values of the cucumber plants that received 700 ppm of CO_2_ under PRD conditions were higher than those of the control plants under full irrigation conditions (Fig. 3).

#### Plant fresh weight

**Fig. 4:**
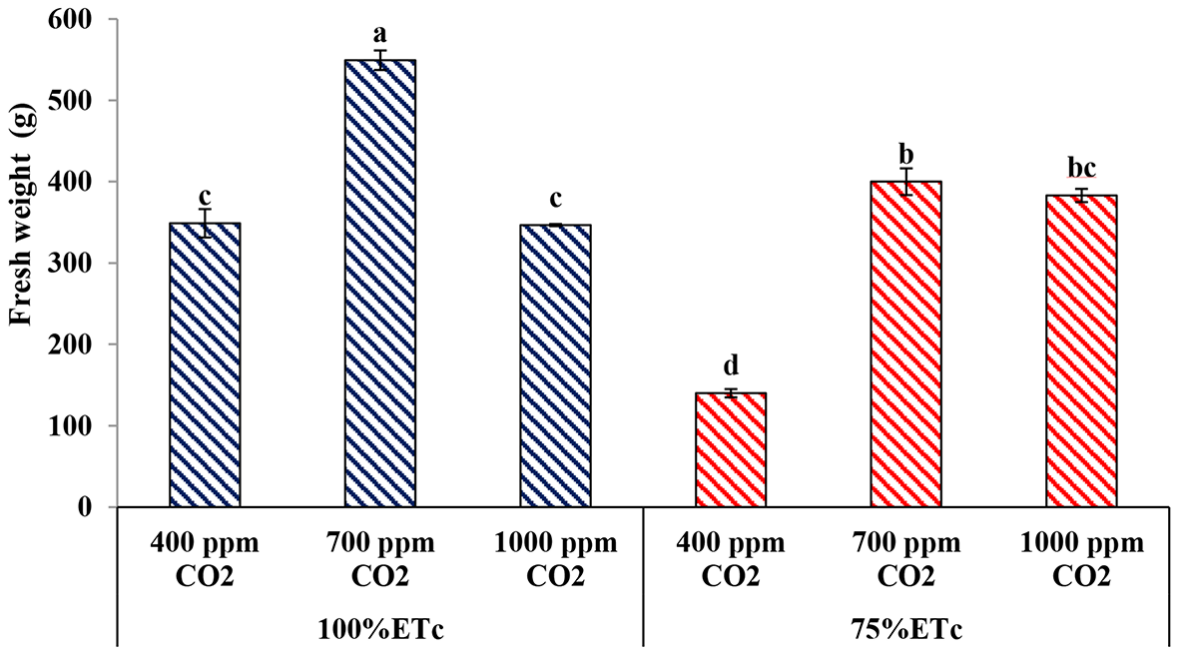
Effect of interaction between water levels and elevated CO_2_ levels on plant fresh weight. Different letters indicate significant differences between treatments (Duncan’s multiple range test at *P* < 0.05)

As expected, PRD caused a reduction in the fresh weight of the cucumber plant more than plants grown under full irrigation conditions (Fig. 4). Under both irrigation conditions, enriched cucumber plants with 700 ppm CO_2_ significantly increased plant fresh weight compared with the control plants. Additionally, under PRD conditions, both CO_2_ concentrations (without a significant difference between them) enhanced the fresh weight of the cucumber plant compared to the control.

#### Plant dry weight

**Fig. 5:**
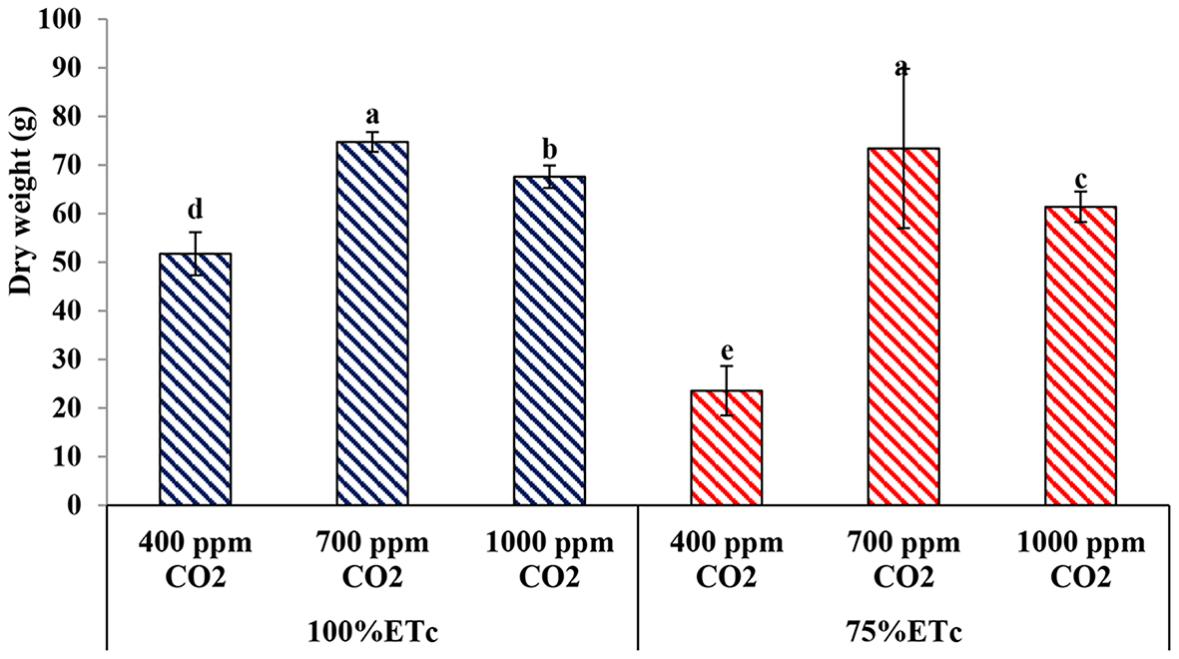
Effect of interaction between water levels and elevated CO2 levels on plant dry weight. Different letters indicate significant differences between treatments (Duncan’s multiple range test at *P* < 0.05)

The dry weight of cucumber plants growing under PRD conditions was less than the dry weight of cucumber plants growing under full-irrigation conditions (Fig. 5). Under both water levels, the treatment of cucumber plants with both levels of CO_2_ led to a significant increase in the dry weight of cucumber plants, Moreover, under drought conditions, enriched plants with 700 ppm and 1000 ppm CO_2_ significantly increased the plant dry weight compared to the control treatment under full irrigation conditions.

#### Number of shoots per plants

**Fig. 6:**
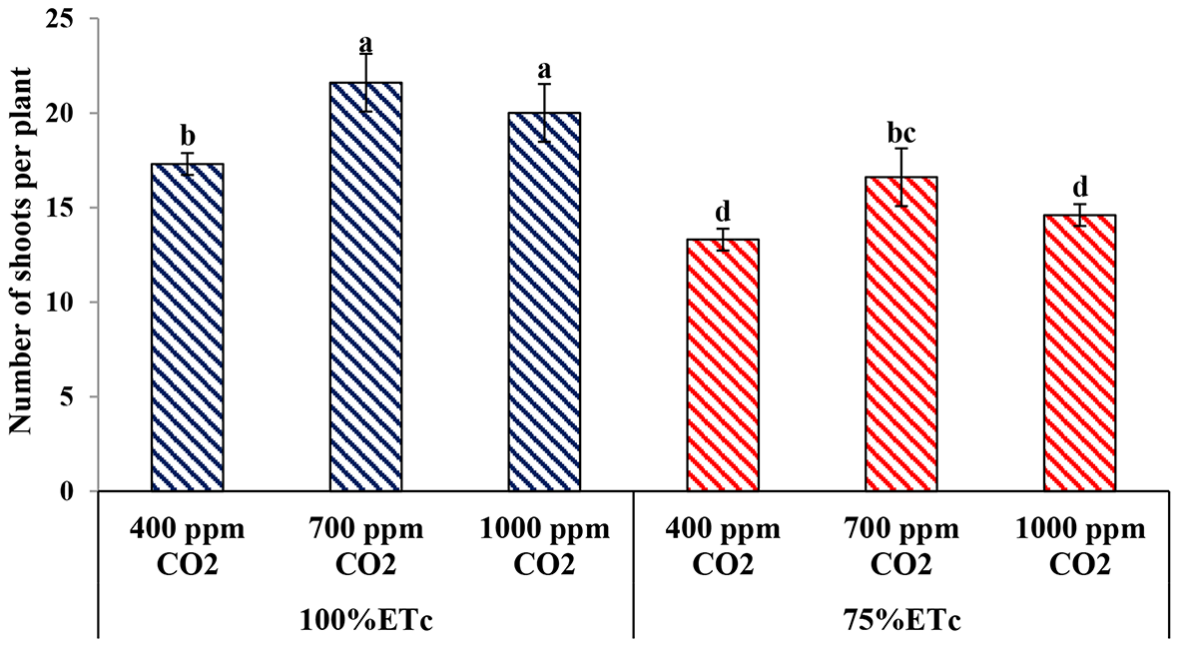
Effect of Effect of interaction between water levels and elevated CO_2_ levels on number of shoots/plant. Different letters indicate significant differences between treatments (Duncan’s multiple range test at *p* < 0.05)

In this investigation, the results of Fig. 6 shows that plants under PRD conditions had a lower number of shoots compared to the plants that received the recommended water quantity. Under full irrigation conditions, enriched plants with 700 ppm or 1000 ppm of CO_2_ significantly increased the number of shoots compared to the control treatment (400 ppm). Under PRD conditions, enriched plants with 700 ppm only increased the number of shoots per plant. While, insignificant differences were observed among untreated plants and enriched plants with 1000 ppm of CO_2_.

#### Leaf area

**Fig. 7:**
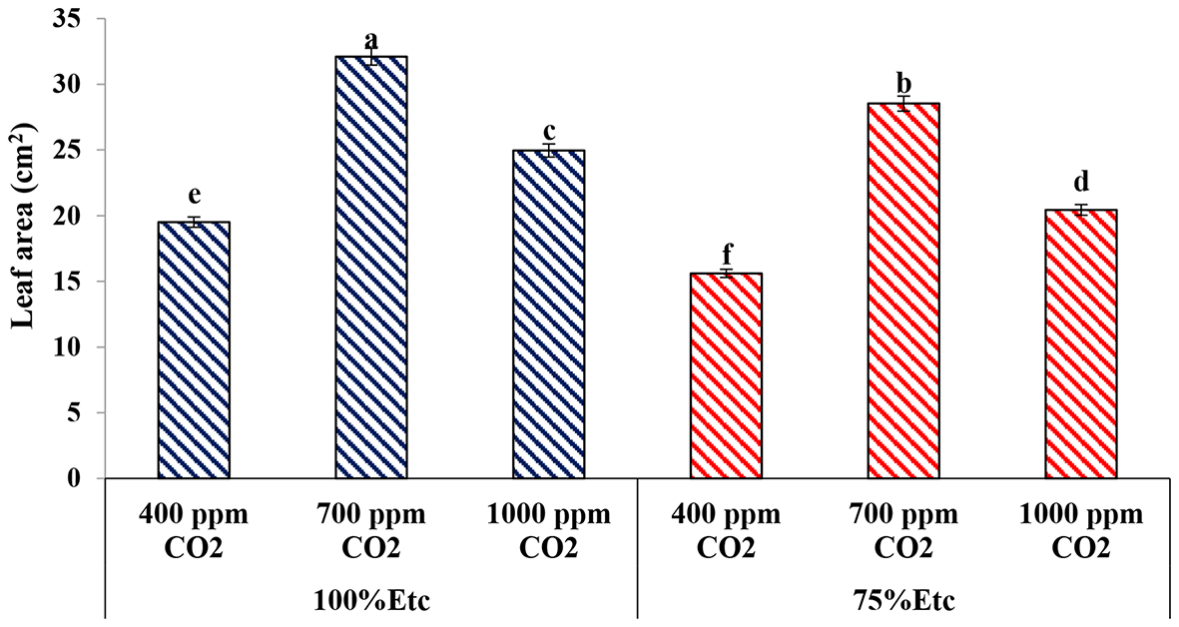
Effect of interaction between water levels and elevated CO_2_ levels on leaf area. Different letters indicate significant differences between treatments (Duncan’s multiple range test at *P* < 0.05)

As shown in Fig. 7, the leaf area (LA) of cucumber plants was influenced by eCO_2_ and PRD. It was observed that PRD caused a reduction in the values of leaf area of cucumber plants than ones grown under full irrigation conditions. Both eCO_2_ levels (700 and 1000 ppm) significantly increased the leaf area of cucumber plants under PRD or full irrigation conditions (*P* ≤ 0.05). However, enriched plants with 700 ppm or 1000 ppm of CO_2_ increased the leaf area of cucumber plants by 39.22% and 21.82% under full irrigation conditions and 45.30% and 23.64% under PRD conditions, respectively, compared with control plants.

#### Root length

**Fig. 8:**
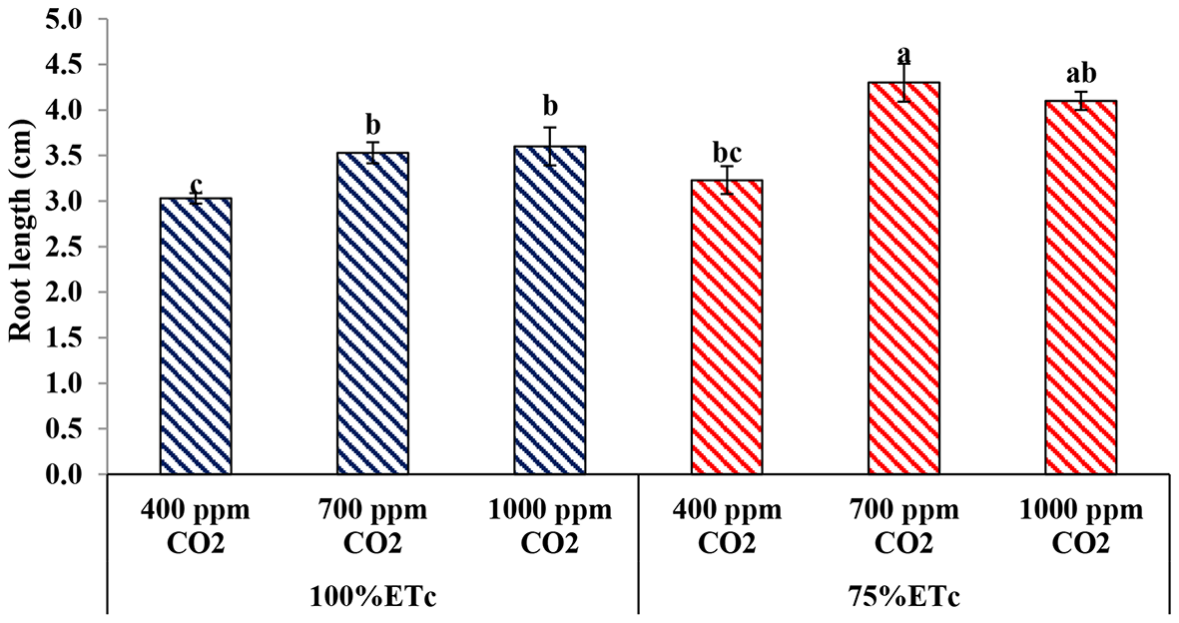
Effect of interaction between water levels and elevated CO_2_ levels on root length. Different letters indicate significant differences between treatments (Duncan’s multiple range test at *P* < 0.05)

In present study, the results of Fig. 8 shows the effect of PRD and enriched soil with two levels of CO_2_ on the root length of the cucumber plant. There was no significant effect of the PRD on root length. However, under both water levels, the application of CO_2_ at doses of 700 and 1000 ppm significantly increased root length without significant difference between both concentrations. Compared to the untreated plants, the improvement ratio in root length reached 14.16% and 15.83% under full irrigation conditions and 24.88% and 21.22% for enriched plants with 700 ppm or 1000 ppm of CO_2_.

### Photosynthesis measurements

Effects of water levels and eCO_2_ levels on the photosynthetic rate, transpiration rate, stomatal conductance, and CO_2_ concentration in the stomata of cucumber plants (Table 5). The photosynthesis rate (*Pn*), transpiration rate (Tr), and stomatal conductance (SC) decreased, while Intercellular CO_2_ concentration increased under PRD conditions compared with full irrigation treatment. The most effective treatment for enhancing photosynthetic rate under either PRD or full-irrigation conditions was 700 ppm CO_2_, followed by 1000 ppm CO_2_, compared with the control. The transpiration rate was decreased by the 700 ppm CO_2_ treatment compared with the control or the higher concentration (1000 ppm) under both water levels. The stomatal conductance increased significantly with the treatment of 700 ppm CO_2_ compared with 400 and 1000 ppm CO_2_ under either full irrigation or PRD conditions (Table 5). The higher CO_2_ concentration (Ci) in the stomata was observed by a 700 ppm CO_2_ application followed by a 1000 ppm CO_2_ treatment under both water treatments.

**Table 5:** Effect of water levels and elevated elevetaed CO_2_ levels on water productivity

ET_C_	CO_2_ concentrations (ppm)	Irrigation Water applied (m^3^ ha^− 1^)	Water productivity (Kg m^− 3^)
100% ET_C_	400	1936.73 a	50.39 b
	700	1936.73 a	59.22 a
	1000	1936.73 a	54.32 ab
75% ET_C_	400	1452.55 b	30.31 c
	700	1452.55 b	55.14 ab
	1000	1452.55 b	51.70 b

Different letters indicate significant differences between treatments (Duncan’s multiple range test at *p* < 0.05)

### Leaf carbohydrate content

**Fig. 9:**
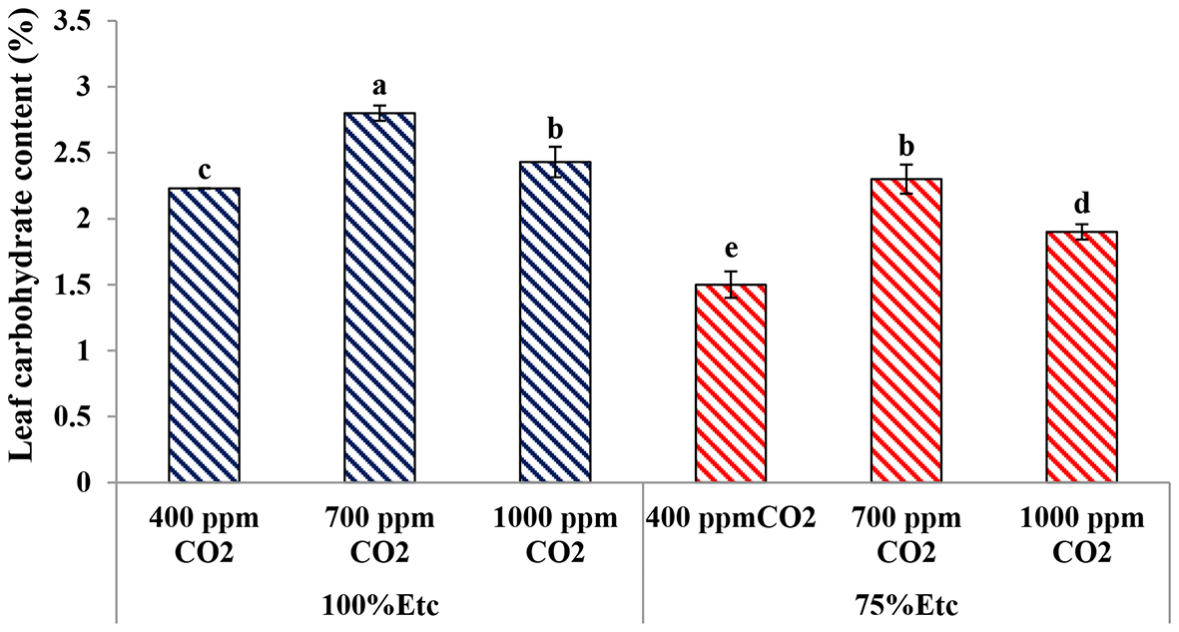
The Effect of interaction between water level and elevated CO_2_ level on leaf carbohydrates content. Different letters indicate significant differences between treatments (Duncan’s multiple range test at *P* < 0.05)

The impact of both water regimes and eCO_2_ levels on leaf carbohydrate content is presented in Fig. 9. The PRD caused a reduction in leaf carbohydrate content compared to the full irrigation conditions. Conversely, eCO_2_ treatment significantly upgraded the leaf carbohydrate content of cucumber plants compared to the untreated plants, under both water levels. Compared with the untreated plants, eCO_2_ at doses of 700 and 1000 ppm increased leaf carbohydrate content by 20.36% and 8.23% at 100% ETc (full irrigation conditions) and 34.78% and 21.05% at 75% ETc (PRD conditions).

### Leaf antioxidant content

**Fig. 10:**
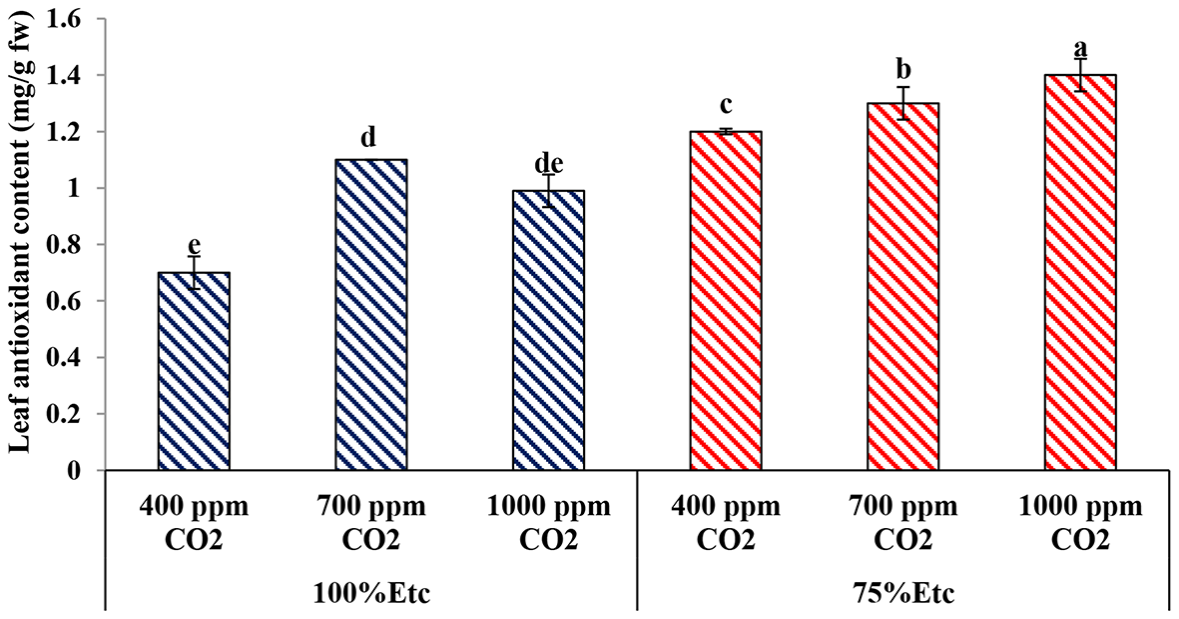
Effect of interaction between water stress and soil CO_2_ concentrations on leaf antioxidant content. Different letters indicate significant differences between treatments (Duncan’s multiple range test at *P* < 0.05)

The leaf antioxidant content in cucumber leaves increased under PRD conditions compared with the full irrigation conditions. Under well-watered conditions, the 700 ppm CO_2_ treatment significantly increased leaf antioxidant content compared to the control, which had no significant difference with the 1000 ppm CO_2_. Additionally, the 1000 ppm CO_2_ treatment recorded the highest leaf antioxidant content, followed by the 700 ppm CO_2_ treatment, while the control (400 ppm CO_2_) recorded the lowest values (Fig. 10).

### Leaf proline content

**Fig. 11:**
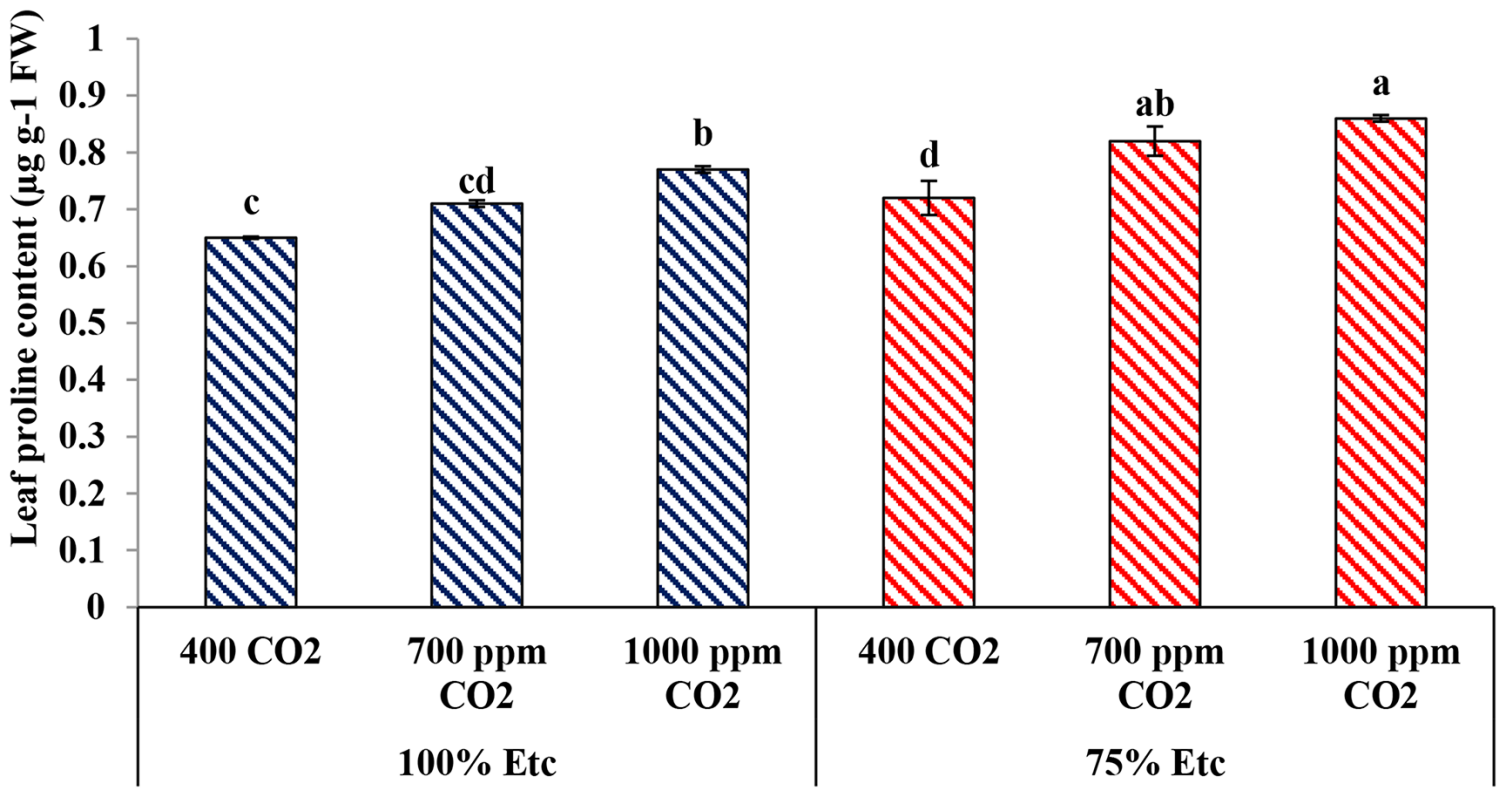
The Effect of interaction between water levels and soil CO_2_ concentrations on leaf proline content. Different letters indicate significant differences between treatments (Duncan’s multiple range test at *P* < 0.05)

The data in Fig. 11 shows that Proline content increased under PRD condition and eCO_2_ levels. Under both water levels, eCO_2_ levels showed higher Proline values than the control treatment (400 ppm CO_2_). Compared with the control, eCO_2_ at a rate of 700 and 1000 ppm improved leaf Proline content by 8.45% and 15.58 at 100% ETc and 12.20% and 16.28 at 75% ETc, respectively. Under eCO2 (1000 ppm) and PRD conditions, the greatest value of Proline content was recorded in leaves of cucumber plants compared to all the other treatments.

### Leaf phytohormones content

**Fig. 12:**
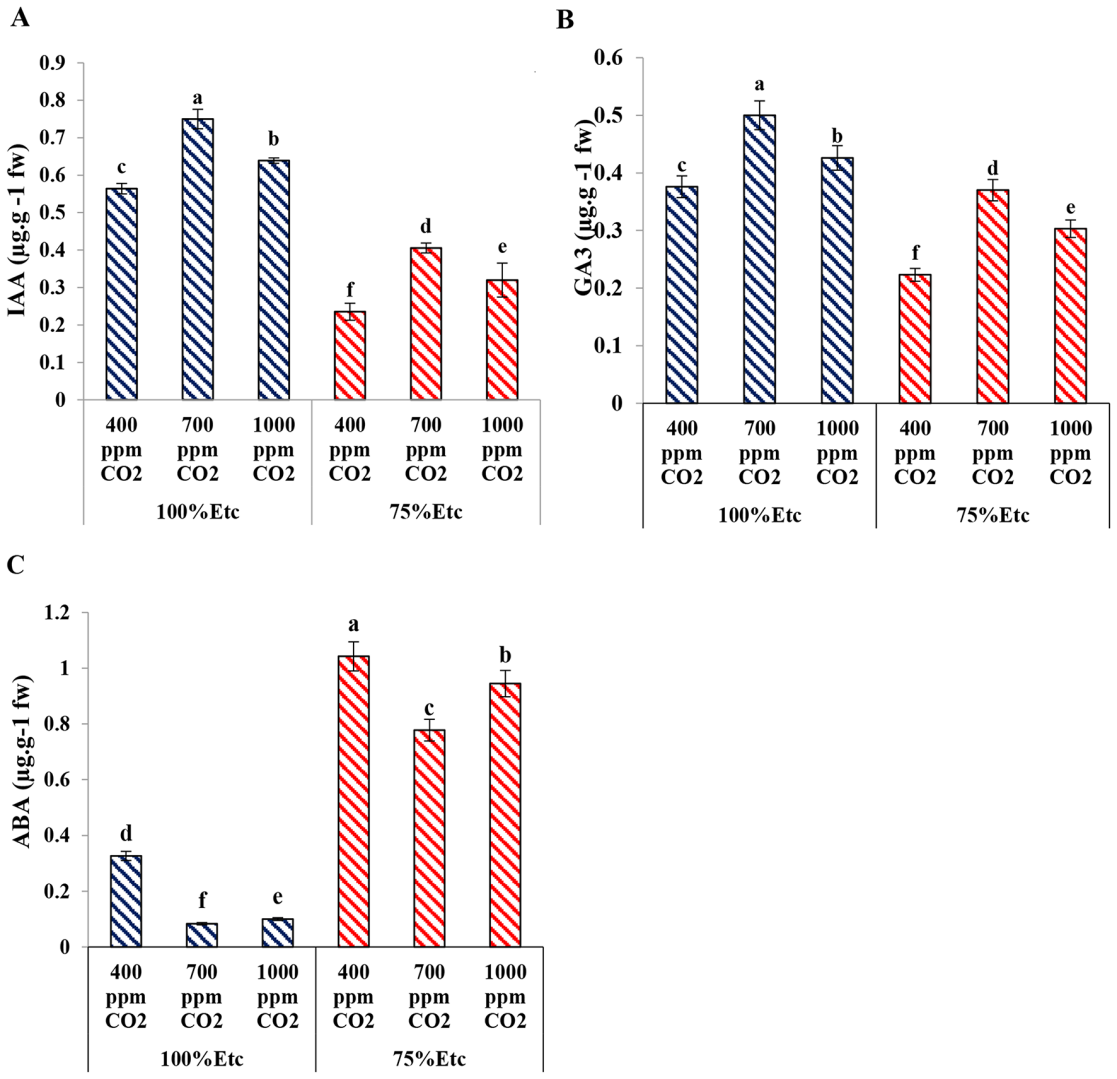
The effect of interaction between water levels and soil CO_2_ concentrations on concentrations of IAA (A), GA3 (B) and ABA (C). Different letters indicate significant differences between treatments (Duncan’s multiple range test at *P* < 0.05)

The effects of PRD and soil CO_2_ concentrations on plant hormones are illustrated in Fig. 12. The IAA content significantly decreased under PRD conditions compared with the full irrigation conditions. Elevated CO_2_ (at 700 ppm) showed the maximum values of leaf IAA under both water levels, followed by 1000 ppm CO_2_ treatment while the lowest values was observed in the control (400 ppm of CO_2_), as presented in (Fig. 12A). Furthermore, the content of GA3 decreased under the PRD condition compared with the full irrigation conditions. In comparison with 4000 ppm CO_2_ (Control), 700 ppm CO_2_ treatment was the most effective treatment for increasing GA3 under full irrigation conditions, followed by 1000 ppm CO_2_ treatment under both irrigation levels (Fig. 12B). On the contrary. The ABA content significantly increased under the PRD condition in comparison with the full irrigation condition. In comparison with 400 ppm CO_2_ (Control), both CO_2_ levels (700 ppm and 1000 ppm) showed higher GA3 levels under both water levels compared to the control (Fig. 12C).

### Leaf nutrient contents

The PRD (75% ETc) significantly decreased the absorption of N, P, K, Ca, and Mg nutrients from the soil compared to full irrigation conditions. Under full irrigation conditions, the 700 ppm CO_2_ treatment was the most effective dose for increasing the content of all nutrients in the leaves of cucumber plants, followed by the 1000 ppm CO_2_ treatment, while the 400 ppm CO_2_ treatment (Control) recorded the lowest values. Moreover, under PRD conditions, 700 ppm CO_2_ treatment significantly increased the contents of minerals compared to the higher concentration or the control treatment (Table 6).

**Table 6:** The effect of water levels and e CO_2_ levels on photosynthesis measurements of cucumber plants

ET_C_	CO_2_ concentration (ppm)	Pn (µmol m^–2^ s^–1^)	Tr (mmol m^–2^ s^–1^)	SC (mmol m^–2^ s^–1^)	Ci (ppm)
100% ET_C_	400	9.51 d	15.83 a	0.196 b	279.3 d
	700	19.23 a	14.26 b	0.283 a	339.6 c
	1000	16.783 b	15.3 a	0.176 b	365 bc
75% ET_C_	400	6.203 e	12.76 c	0.166 bc	372.6 bc
	700	12.576 c	12 d	0.196 b	424.3 a
	1000	11.95 c	12.53 c	0.136 c	388 ab

*Pn* = photosynthesis rate, SC = stomatal conductance, Tr = transpiration rate, and Ci = intercellular CO_2_ concentration

Different letters indicate significant differences between treatments (Duncan’s multiple range test at *p* < 0.05)

### *Yield and yield components*

The effect of PDR and soil eCO_2_ levels on yield and its components in cucumber plants (Table 7). As expected, yield and its parameters significantly decreased under PRD condition compared to the Full irrigation conditions. The fruit weight of cucumber fruits was not significantly affected by all CO_2_ concentrations under full irrigation conditions. The 1000 ppm of CO_2_ treatment was the most efficient treatment for enhancing the fruit weight of cucumber fruits under PRD conditions. The number of fruits per plant increased significantly by both CO_2_ concentrations under both water levels, and the superior concentration was 1000 ppm, followed by 700 ppm CO_2_. Similarly, the total yield per plant, early yield, and the total yield per ha increased significantly by both CO_2_ levels under both water levels.

**Table 7:** The effect of water levels and soil CO_2_ concentrations on leaf nutrient content of cucumber plants

ET_C_	CO_2_ concentration (ppm)	*N* (%)	*P* (%)	K (%)	Ca (%)	Mg (%)
100% ETC	400	4.5 c	1.03 b	4.1 b	6.1 b	0.64 c
	700	4.7 a	1.018 bc	4.3 a	6.53 a	0.67 a
	1000	4.6 b	1.08 a	4.16 ab	6.2 b	0.66 b
75% ET_C_	400	4.3 e	0.9 e	3.9 c	5.36 d	0.606 e
	700	4.43 d	1.003 d	4.06 b	5.73 c	0.63 cd
	1000	4.3 e	0.98 cd	4.03 bc	5.5 d	0.623 d

Different letters indicate significant differences between treatments (Duncan’s multiple range test at *p* < 0.05)

### Fruits quality and chemical compositions

The effect of PRD and soil CO_2_ concentrations on TSS, hardness, carbohydrate content, Proline, antioxidant content, and minerals (N, P, K, Ca, Mg) of cucumber plants is shown in Tables 6 and 7. All fruit quality variables were significantly affected by the water levels and CO_2_ concentrations. Additionally, under both water conditions, enriched soil with 700 and 1000 ppm CO_2_ significantly enhanced TSS content in cucumber fruits in comparison with the control. The plants that were grown under the 75% ETc condition produced fruits significantly firmer than those plants that received 100% ETc when the soil was enriched with 700 or 1000 ppm CO_2_ (Table 8).

**Table 8:** The effect of water levels and elevated CO_2_ levels on yield and its components of cucumber plants

ET_C_	CO_2_ concentration (ppm)	Average fruit weight (g)	Number of fruits/plant		Total yield/plant (Kg)	Early yield (ton ha^− 1^)	Total yield (ton ha^− 1^)
100% ET_C_	400	61.0 ab	57.0 c	3.48 b		3.85 c	97.6 c
	700	63.3 a	71.0 a	4.09 a		5.58 b	114.7 a
	1000	62.6 a	60.0 b	3.75 ab		7.33 a	105.2 b
75% ET_C_	400	57.3 c	27.0 f	1.57 d		2.55 d	44.02 e
	700	59.6 bc	48.0 d	2.86 c		6.06 b	80.1 d
	1000	63.0 a	43.0 e	2.68 c		5.44 b	75.09 d

Different letters indicate significant differences between treatments (Duncan’s multiple range test at *p* < 0.05)

In comparison with the control, both CO_2_ concentrations significantly increased the hardness of the cucumber fruits at both water levels. Carbohydrate content was significantly decreased at PRD condition compared to the full irrigation conditions in the control treatments (400ppm CO_2_) (Table 8). Additionally, there were no differences between water treatments and CO_2_ concentrations on carbohydrate content. The effect of water treatments and CO_2_ levels on the Proline content of cucumber fruits was not significant or had little effect (Table 8). In general, the antioxidant content increased with PRD condition compared to the control (400 ppm CO_2_). Enriched soil with 700 ppm CO_2_ was the most positive treatment to enhance antioxidant content in the cucumber fruits, either under PRD or under full irrigation conditions.

The data in Table 9 shows that uptake per cucumber plant of N, P, K, Ca, and Mg was greater under full irrigation conditions than PRD conditions in all CO_2_ concentrations. The concentration of N increased with the 700 ppm CO_2_ treatment, followed by a higher concentration compared to the control under both water conditions. Under drought stress, no CO_2_ treatments affected the concentration of P, while under well-watered conditions, both CO_2_ concentrations increased P content compared to the control. The concentration of K and Ca increased by both CO_2_ levels (without significant difference between them) compared to the control under both water levels. The effect of water levels and soil CO_2_ concentrations on the Mg content of cucumber fruits was not significant.

**Table 9:** The effect of water levels and soil CO_2_ concentration on fruit quality of cucumber plants

ET_C_	CO_2_ concentration (ppm)	TSS (Brix)	Hardness (kg cm^− 2^)	Carbohydrate content (%)	Proline content (µg g^− 1^ FW)	Antioxidant content (mg g^− 1^ FW)	
100% ET_C_	400	5.03 c	2.53 d	20.10 b	0.40 b	1.91 d	
	700	5.63 ab	3.63 b	25.02 a	0.43 ab	2.60 b	
	1000	5.33 b	3.2 c	24.60 ab	0.40 b	2.19 c	
75% ET_C_	400	5.06 c	2.6 d	17.01 c	0.46 ab	2.34 bc	
	700	5.93 a	4.2 a	23.01 abc	0.50 a	2.80 a	
	1000	5.73 ab	4.03 a	22.30 bc	0.51 a	2.77 a	

Different letters indicate significant differences between treatments (Duncan’s multiple range test at *p* < 0.05)

### Expression of aquaporin-related genes (CsTIP4 and CsPIP1-2) and stress-responsiveness Argonaut (CsSAGO1) in cucumber plants under water deficit stress conditions

**Fig. 13:**
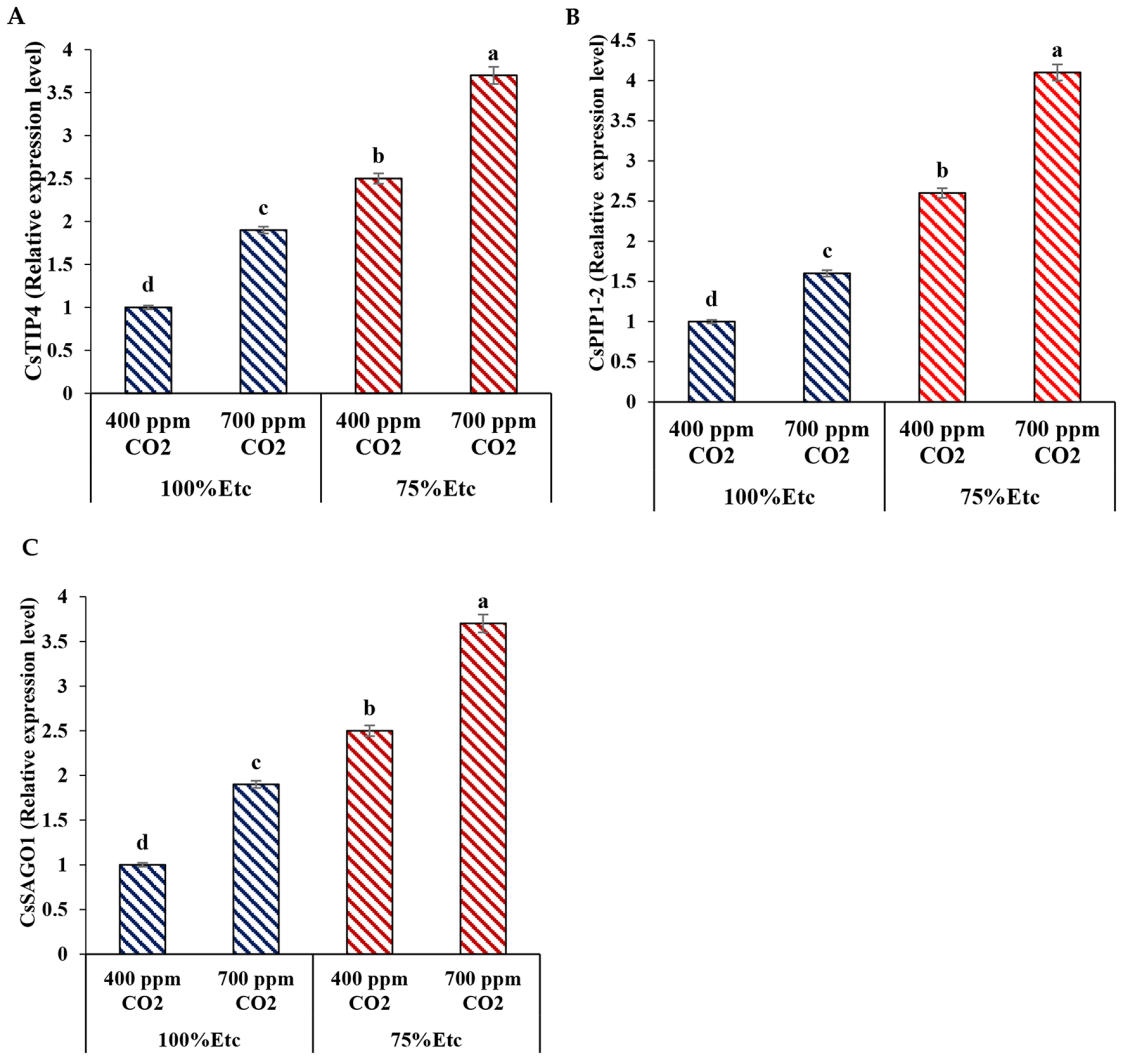
Relative expressions level of *CsTIP4* (A), *CsPIP1-2* (B) and *CsSAGO1* (C) genes of the treated cucumber with carbon dioxides under partial root-zone drying (PRD) and full irrigation conditions .Different letters indicate significant differences between treatments (Duncan’s multiple range test at *P* < 0.05)

The mechanism of CO_2_ enrichment regulation of water equilibrium via modulating aquaporin-related (PIP and TIP) and stress-responsiveness Argonaut (*CsSAGO1*) genes in plants was investigated under PRD condition (water stress). This discovery gives us an understanding of how CO_2_ enhancement improves water stress in cucumber. The transcript levels of aquaporin-related genes and stress-responsive genes in cucumber leaves under different water deficit severities and concentrations of CO_2_ and the transcription of aquaporin-related TIP genes (CsTIP4) and PIP genes (*CsPIP1-2*) in the cucumber plants were investigated (Fig. 13A and B).

Notwithstanding, high CO_2_ concentrations and water deficit stress could significantly upset the transcript levels of all two genes. While this study revealed that the transcript levels of the CsTIP4 gene amplified increased significantly in plants treated with 700 ppm CO_2_ under a 75% water deficit degree, in this respect, the expression of aquaporin-related genes (CsPIP1-2) has also significantly increased in plants treated with 700 ppm CO_2_ under water deficit stress. Aquaporin-mediated cell-to-cell pathways are the major pathways of water transport in seedling roots under CO_2_ and water treatments.

On the other hand, the transcript levels of stress-responsiveness Argonaut (CsSAGO1) were carried out by real-time PCR in treated plants with 700 ppm and control plants during water deficit stress (Fig. 13C). The results verified that the *CsAGO1* transcript level gene in control plants showed no significant variation under normal conditions, whereas transcript CsAGO1 gene levels were upregulated in 700 ppm-treated plants but down regulated in control plants (non-treated with 700 ppm).This gene’s mRNA levels were higher in treated than control plants. As a result, CsAGO1 transcript levels increased by 3.6 fold in treated plants with eCO2 at 700 ppm. While the CSAGO1 transcript levels decreased to 1.2 fold in control under PRD condition. The results confirmed that CO_2_ supplementation developed water deficit tolerance and promoted the tolerance of cumber plants to water deficit stress.

### Correlation study

**Fig. 14:**
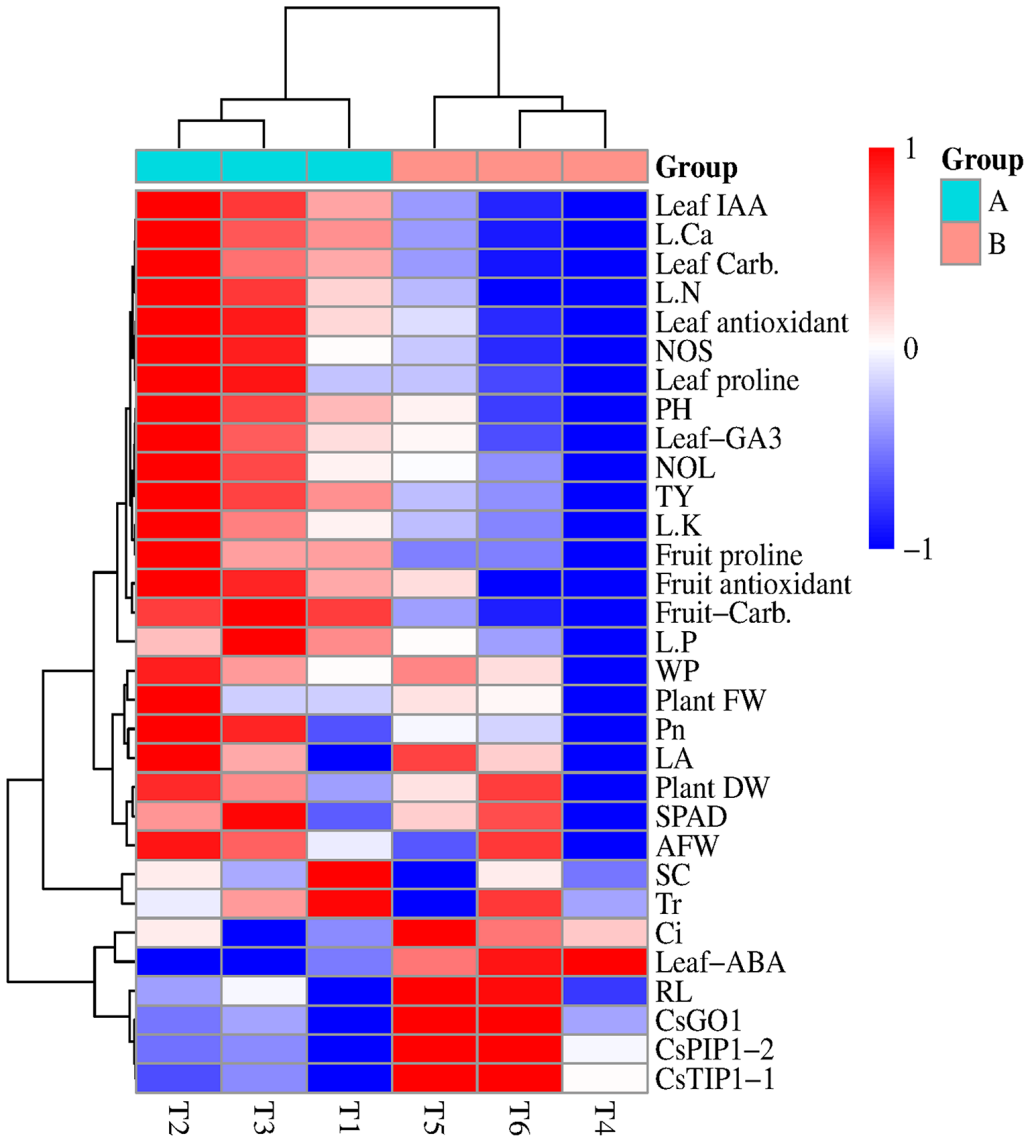
Heatmap correlation between Agro-physiochemical and genetically parameters of cucumber plants grown under normal and stress conditions and treated with carbon dioxide (700 ppm and 1000ppm). *Abbreviations* RL, root length; NOL, number of leaves; PH, plant height; LA, leaf area; FW, plant fresh weight; DW, plant dry weight; EY, early yield; IAA, indole-3-acetic acid; GA3, gibberellic acid; ABA, Abscisic acid; *Pn*, photosynthesis rate; SC, stomatal conductance; Ci, intercellular CO_2_ concentration; AFW, average of fruit weight; NOF, number of fruit; LAC, leaf antioxidant content; LCB, leaf carbohydrate; FCB, fruit carbohydrate; and TY, total yield

**Fig. 15:**
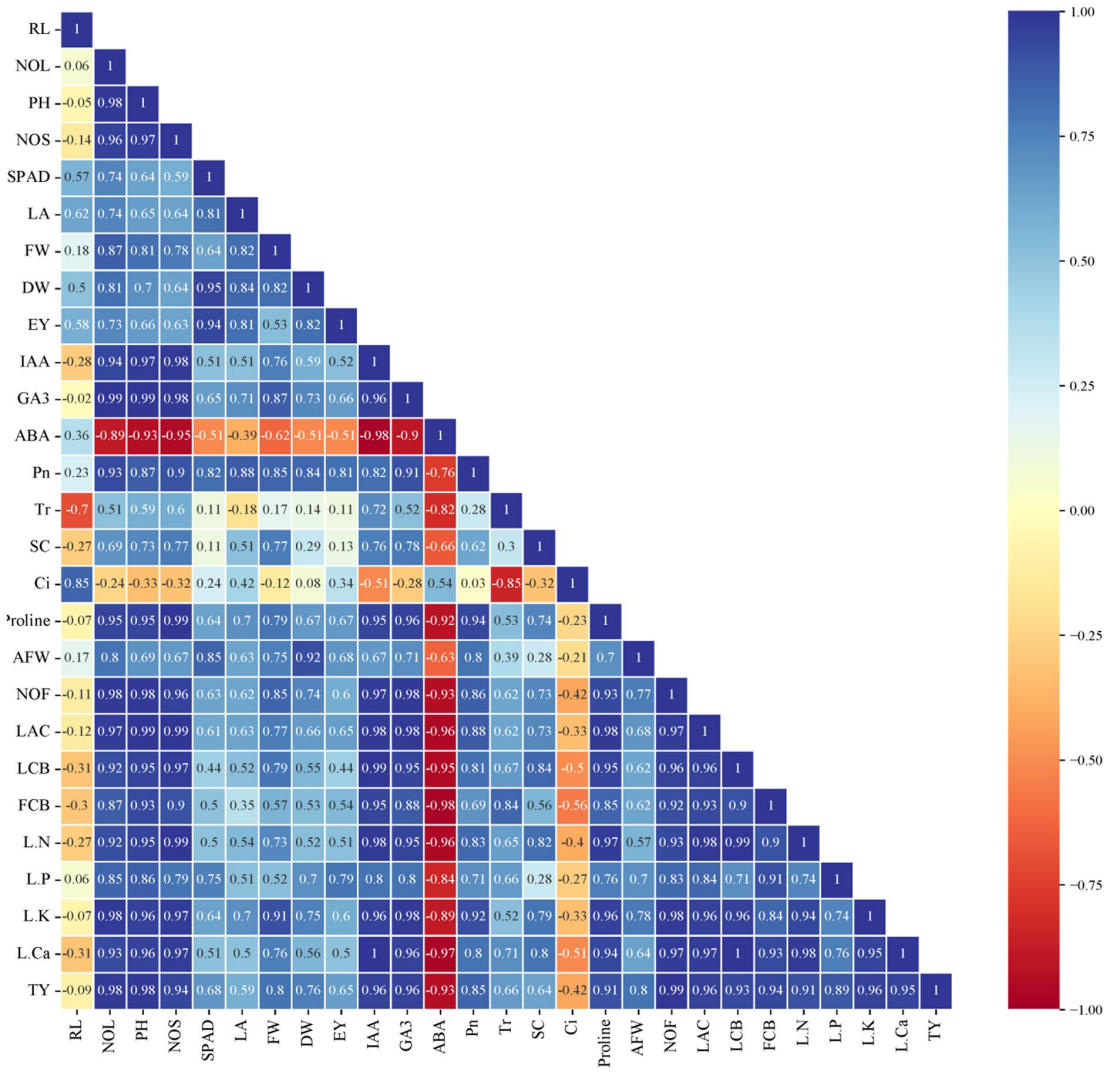
Pearson’s correlation analysis between agro-physiochemical and biochemical properties of cucumber plants exposed to different concentration of soil carbon dioxide under different levels of irrigation. *Abbreviations* RL, root length; NOL, number of leaves; PH, plant height; LA, leaf area; FW, plant fresh weight; DW, plant dry weight; EY, early yield; IAA, indole-3-acetic acid; GA3, gibberellic acid; ABA, Abscisic acid; Pn, photosynthesis rate; SC, stomatal conductance; Ci, intercellular CO_2_ concentration concentration; AFW, average of fruit weight; NOF, number of fruit; LAC, leaf antioxidant content; LCB, leaf carbohydrate; FCB, fruit carbohydrate; and TY, total yield

Pearson’s correlation analysis and Heatmap correlation show the alterations in agro-physicochemical, biochemical, and genetic assets of cucumber plants exposed to different soil carbon concentration under different irrigation levels (Figs. 14 and 15). Furthermore, the Heatmap correlation showed that eCO_2_ has positive impacts on morphological properties and enhanced most of the vegetative growth parameters of cucumber plants under full irrigation (100% ETc) and PRD conditions (75% ETc).

The Heatmap based on the 31 measurements clearly classified them into two groups (A and B), while the cucumber grown under full irrigation condition (100% ETc) treated with carbon dioxide at 700ppm (T2),1000ppm (T3) and untreated plants (T1 = Control / 400 ppm of CO_2_) were inserted together under group A. Meanwhile, the second group (B) included the cucumber grown under PRD conditions (75% ETc) treated with carbon dioxide at 700ppm (T5),1000ppm (T6) and untreated plants (T4 = control or 400 ppm). The red color indicates a positive effect, and the blue color presented a negative effect. The Heatmap correlation also indicates that the treatment T2 (700ppm CO_2_ + 100% ETc) and T3 (1000ppm CO_2_ + 100% ETc) have more positive effect on the most of selected measurements except for transpiration rate (Tr), stomatal conductance (SC), intercellular CO_2_ concentration (Ci); relative expression genes (CsTIP4, CsPIP1-2 and CsSAGO1). While the treatment T5 (700ppm CO_2_ + 75% ETc) and T6 (1000ppm CO_2_ + 75%ETc) have positive impact on relative expression genes (CsTIP4, CsPIP1-2 and CsSAGO1).

Likewise, Pearson’s correlation analysis was used to identify the positive and negative correlations between the studied parameters. A positive correlation (blue color) and negative relationships (red color) (Fig. 15). Pearson’s correlation analysis exhibited that total yield positively associated with root length, number of leaves, plant height, number of shoots, leaf area, shoot fresh weight, shoot dry weight and early yield. A similar correlation also was noted in total yield and indole-3-acetic acid, gibberellic acid, photosynthesis, average fruit weight, number of fruits, leaf antioxidant content, leaf carbohydrate, and fruit carbohydrate. On the other side, the total yield correlated negatively with Abscisic acid.

## Discussions

Water and carbon dioxide (CO_2_) are considered the most important factors affecting crop growth, and ongoing climate change affects water and atmospheric CO_2_, which impacts crop production. In the present study, we investigated the effect of the water-saving irrigation technique affected the plant performance, photosynthesis apparatus, water productivity, and quality of cucumber grown under elevated CO_2_ conditions [5, 10].

Numerous reports suggest that eCO_2_ levels and water deficit stress significantly affect agrophysiological properties and productivity of cash crops [51]. Elevated CO_2_ (eCO_2_) stimulates the accumulation of secondary compounds, regulates secondary metabolism, enhances adaptability, photosynthesis, and net assimilation rate, thereby improving crop yield [52, 53]. In addition, few researches have been demonstrated the impacts of combinations of eCO_2_ levels and other ecological factors, including high temperature, light intensity, and water shortage, on the growth of many crops [3, 23, 51] However, the long-term effects of the eCO_2_ and treatments on cucumber plants grown under water deficit stress (drought stress) still remain unclear.

Several previous studies have demonstrated the effects of drought stress conditions on cucumber vegetative growth and reported that drought stress detrimentally affects plant growth [12, 15, 17, 19]. In the current study, drought stress significantly reduced the vegetative growth measurements of cucumber plants, namely; plant height, number of leaves, number of shoots, leaf area, and plant biomasses (Figs. 1, 2, 3, 4, 5, 6, 7 and 8). This reduction in plant growth parameters such as leaf area and number of leaves could be one of the plant strategies to tolerant drought stress by reducing evaporation from the surface area [54]. The reduction in leaf area under water stress conditions was also reported by previous investigations [55]. Meanwhile, elevated CO_2_ (700 ppm) augmented the leaf area and plant biomass (fresh and dry weight) in both full irrigation (100% ETc) and moderate water stress conditions (PRD, 75% ETc). In agreement, Liu and Stützel [54] reported that elevated CO_2_ concentration significantly increased leaf area and plant biomasses of cucumber plants. Similar findings were observed by Leibar-Porcel et al. [56] who confirmed that the number of leaves and dry biomass of lettuce was 22% higher than the control treatment under elevated root zone CO_2_. Furthermore the Heatmap correlation showed that eCO_2_ has positive impacts on morphological properties and enhanced most of the vegetative growth parameters of cucumber plants under full irrigation (100% ETc) and PRD conditions (75% ETc), as shown in Fig. 14.

These improvements in number of leaves, leaf area, fresh biomass, and dry biomass of cucumber plants could be more associated with increasing the photosynthesis rates which can manufacture and accumulate carbohydrates, laying the basis for the development of the leaf structures and increment of plant biomasses [53, 57]. Under moderated drought stress (75% ETc), the root morphology changed (Fig. 8) to cope with the water shortage [58, 59]. In this study and the previous study [20] confirmed that enriching the soil with CO_2_ improves root length which increases the ability of plants to absorb additional amount of water and nutrients [60]. Additionally, increasing plant growth by CO_2_ treatment could be due to the increasing of plant growth hormones including IAA and GA3 [61, 62] which recorded in our study (Fig. 12A&B). These findings also indicated that eCO_2_ partially mitigates the adverse effects of moderate water stress on the vegetative growth of cucumber plants [63].

As presented in Fig. 3; Table 5, chlorophyll content (SPAD value), photosynthetic rate, and stomatal conductance of cucumber leaves decreased under PRD stress conditions. This result might be due to the stomatal and non-stomatal photosynthetic limitations under drought stress conditions [54], the increased activity of the enzymes responsible for chlorophyll degradation [61], and the detrimental effects of reactive oxygen species (ROS) on chloroplasts, which reduced carbon assimilation [56]. A previous work recorded a reduction in stomatal conductance of *Jatropha curcas* under drought stress ranging from 100 − 25% field capacity [64]. Conversely, in this study elevated CO_2_ in soil increased the content of leaf chlorophyll and improved photosynthetic rate (Pn), stomatal conductance (SC), and intercellular CO_2_ concentration (Ci). Besides, Pearson’s correlation analysis confirmed that chlorophyll content (SPAD) correlated positively with photosynthesis rate (Pn). Several studies have revealed that eCO_2_ upsurges the net photosynthetic rate (Pn) in various C3 crops by improving intercellular CO_2_ concentration (Ci), increasing carboxylation efficiency, and declining photorespiration [65].

Signaling phytohormones such as ABA, IAA, and GA3 are related with the regulation of plant biochemistry processes especially under stresses [66]. In this study, under both water levels (100 ETc and 75% ETc), the application of eCO_2_ (700 ppm and 1000 ppm) significantly improved the levels of IAA and GA3 and reduced ABA level compared with the control (400 ppm CO_2_), as shown in Fig. 12 (A, B, C). These findings agree with some previous studies on cucumber [67] and tomatoes [57]. ABA hormone is classified as a plant growth inhibitor [68] therefore; it increases under drought stress [69]. This result may be because the treatment of CO_2_ leads to a decrease in the activity of peroxidase [63], which in turn leads to a decrease in the decomposition of the IAA [70]. Additionally, it has been found that elevated CO_2_ enhanced the levels of IAA and GA3 in the Ginkgo leaves [69]. A previous study also observed that CO_2_ treatment enhanced calcium concentration, which enhanced the synthesis of GA3 [71].

Both water levels and eCO_2_ concentrations affected leaf and fruit chemical compositions, particularly, total carbohydrates, antioxidant content, Proline concentration and nutrient content (Figs. 9, 10 and 11; Tables 3 and 4–5). Several previous studies confirmed the findings of the current study, whereas water deficit stress reduced the accumulation of carbohydrate and nutrient content in the tissue of cucumber leaves and fruits [72]. Conversely, the water limitation increases the content total antioxidant and Proline content in plants, as source defense, to reduce the ROS activity [71].

In this study, enrichment of CO_2_ in soil improves the nutrient uptake and accumulation of carbohydrate, Proline, and antioxidant content under full irrigation (ETc 100%) and PRD conditions (75% ETc) compared to untreated plants. These improvements could be related to the enhancement of photosynthetic rate (*Pn*), carbon assimilation, and stomatal conductance, which led to enhanced nutrient uptake from the soil, and increased metabolites accumulation in leaf and fruit tissues of drought-stressed plants. Meanwhile, Pearson’s correlation analysis showed that *Pn* correlated positively with nutrient accumulation (N, P, K and Ca), carbohydrate, Proline, and antioxidant content (Fig. 15). These results were consistent with the findings reported by Mahmoud et al. and Abdelaziz et al. [50, 72] that eCO_2_ application improved the photosynthesis rate and nutrient accumulation in plant tissues. Leibar-Porce et al. [56] found that enriched root zone CO_2_ enhanced shoot P and N content of lettuce compared to the control (untreated plants).

Regarding water productivity and yield quantity, the present study showed that eCO_2_ levels and PRD, alone or in combination significantly affect water productivity and fruit quality (Table 10). The WP is defined as the percentage of fruit yield to applied irrigation water. In the estimated water scarcity and elevated CO_2_ condition, it is essential to upsurge the WP of crops in general and cucumber yield in particular. The eCO_2_ or water stress has been presented to improve WP in some C3 crops [57]. However, the correlation between WP, and water stress, for cucumber plants has been unknown [69]. In the present study, eCO_2_ improved WP for both water levels (100% ETc and 75% ETc). Under eCO_2,_ the increase in fruit yield was greater in full irrigation and PRD conditions than in untreated plants (Table 2). However, elevating eCO_2_ in soil caused a reduction in irrigation water input at 100% ETc and 75% ETc (Fig. 1). In agreement, Kumar et al. [73] confirmed that the eCO_2_ significantly increased the WP under different water regimes. This improvement is significantly linked to increased water-use efficiency and reduced evapotranspiration [74, 75].

**Table 10:** The effect of water levels and soil CO_2_ concentrations on nutrient content of cucumber fruits

ET_C_	CO_2_ concentration (ppm)	*N* (%)	*P* (%)	K (%)	Ca (%)	Mg (%)
100% ET_C_	400	0.94 c	0.225 c	2.90 bc	0.45 c	0.12 ab
	700	1.10 a	1.02 a	3.31 a	0.75 a	0.14 a
	1000	1.006 b	0.99 b	3.10 ab	0.59 b	0.10 b
75% ET_C_	400	0.77 f	0.074 d	2.39 d	0.085 e	0.103 ab
	700	0.856 d	0.109 b	2.74 c	0.32 d	0.106 ab
	1000	0.84 e	0.098 c	2.69 c	0.12 e	0.08 b

Different letters indicate significant differences between treatments (Duncan’s multiple range test at *p* < 0.05)

Furthermore, cucumber yield and quality are influenced by water levels, elevated CO_2_ concentration, and temperature worldwide. In this study, under eCO_2_ levels, there was an improvement in fruit yield and a reduction in irrigation water input resulting in higher WP. In addition, total soluble solids (TSS), firms, and nutritional values of cucumber fruits increase due to eCO_2_ and PRD treatments. It has been observed that CO_2_ enrichment raises the TSS in some crops such as tomatoes [59, 64], producing fruits with a higher sugar content. In addition, previous studies showed that treatment with CO_2_ may lead to an increase in the hardness of tomato fruits [13]. The results of these previous studies agree with the results of this study that an increase in the concentration of CO_2_ leads to a significant increase in the TSS and hardness of cucumber fruits as shown in Table 4. Nutritional values including, carbohydrate and total antioxidants are essential for human health [76]. The upsurge in carbohydrate and antioxidants in cucumber fruits as a result of treatment with elevated CO_2_ may be due to an increase in the process of photosynthesis, as previously mentioned [71].

Aquaporin-related genes and stress-responsiveness genes are very important Signaling substances are included in the regulation of root hydraulic conductivity and aquaporin’s under drought stress. In this respect, the previous studies have revealed that controlling the aquaporin seems to be the best opportunity when hydraulic conductivity requirements to be altered in the short term [64]. Li et al. [20] stated that the effects of CO_2_ enrichment on the transcript levels of aquaporin-related genes in the cucumber seedlings under drought stress. Ding et al. [65] reported that there is a relationship between the contribution ratio of water uptake and the cell-to-cell pathway (intervened through aquaporin) in cucumber roots when treated with CO_2_ concentration under water deficit stress; consequently, the cucumber seedling roots mostly charity the cell pathway for water absorption. Underneath atmospheric CO_2_ and CO_2_ enhancement, drought stress changed the contribution ratio of cell pathways to water absorption to a variable degree.

Qian et al. [77] observed complex changes in the transcription of aquaporin-related genes under different water deficit stresses and CO_2_ absorptions. Indicated that the water deficit stress caused a rise in the transcript levels of CsPIP and CsTIP under the two CO_2_ concentrations. In this respect, our results revealed that the upregulation transcripts of these genes might characterize a regulatory mechanism to return and reward water uptake in cucumber plants. Signifying that this CsPIP1-2 and CsTIP4 and show significant role in mediating roots water transportation and those weakening in expression may be a significant factor in the reduction in root hydraulic conductivity of the cucumber seedlings. With improvement of the CO_2_ concentration, the transcription of CsPIP improved significantly under the same severity of water stress. It has been established that the regulation of aquaporin gene expression fundamentally contributes to alterations in the hydraulic conductivity of plants under CO_2_ supplementation [75, 78], so the intensification of CsPIP2-7 transcription level may be one of the ways of CO_2_ supplementation to improve water stress.

However, the strength of root hydraulic conductivity is strong-minded by the number and aquaporin function, and the level of mRNA does not suggest the aquaporin activity. The aquaporin activity can regulate by subcellular localization, protein degradation or the change of gating level [79, 80]. Therefore, although transcription certain aquaporin genes increased under drought stress, the drought stress still directed to the reduction of absolute assessment of the hydraulic conductivity of cucumber roots. Our results were similar to the results demonstrated by Ding et al. [81], that is, the aquaporin contribution to root hydraulic conductivity is 60–85%. Under atmospheric CO_2_ and CO_2_ supplementation, moderate and severe water deficits changed the contribution rate of cell pathways to water absorption to variable degrees. It supplementary reveals the opportunity that CO_2_ enhancement acclimatizes to water deficit stress by varying root aquaporin activity or disturbing aquaporin synthesis in the roots [82]. The results reveal a significantly increased in the transcription of aquaporin-related genes under water stress and CO_2_ absorptions (Fig. 13A). Water deficit stress caused a rise in the transcript levels of CsTIP4 under the two CO_2_ concentrations.

The up-regulation of transcripts may characterize a regulatory mechanism to return and reward water uptake in roots [77]. Indicating that these CsPIPs play a noteworthy role in mediating root water transportation and that their weakening in expression may be a significant factor in the reduction in root hydraulic conductivity of the cucumber seedlings. With the improvement of the CO_2_ concentration, the transcription of CsPIP improved significantly under the same severity of water stress. It has been established that the aquaporin gene expression regulation fundamentally contributes to alterations in the hydraulic conductivity of plants under CO_2_ supplementation [75], so the intensification of the CsPIP2-7 transcription level may be one of the ways CO_2_ enhancements can improve water stress. However, the number and function of aquaporin determine the strength of root hydraulic conductivity, and the level of mRNA does not suggest aquaporin activity. Aquaporin activity can be controlled by subcellular localization, degradation of proteins, and an alteration in gating level [79, 80]. Therefore, although certain aquaporin gene transcription improved under water deficit stress, the stress was still directed at plummeting the absolute value of the hydraulic conductivity of cucumber roots.

Additional studies on cucumber treated with CO_2_ confirmed that the stress-responsive genes were stimulated, resulting in enhanced drug tolerance in plants. Based on these results, the drought stress-responsiveness Argonaut gene (CsAGO1) was set up to be higher in treated cucumber plants than in non-treated plants. These genes (CSAGO1) are the main mechanisms of a series of siRNAs used to improve plant growth and tolerance to water stress [82, 83]. Therefore, Zhang et al. [79] indicated that the gene transcript expression levels of AGO in plants were expressed under water stress treatments, providing insights into stress tolerance mechanisms and other physiological processes. Yang et al. [84] indicated that increasing the OsAGOs gene outcomes in response to light and dark usages. Shao and Lu [85] presented that the SmRDRs gene has the greatest improved expression in plants and the lowest regulatory expression levels in flowers in response to water stress.

The treated plants with CO_2_ expressed the oxidase 1 (ACO1) silencer gene, which controls developing stress responses and chromatin structure through protective membrane reliability and proficiently persistent water, both of which are essential procedures for plants to defend plant growth under water stress. Gan et al. [83] found that high expression of Dicer-like (DCL) and Argonaut (AGO) genes leads to RNA silencing inflection to stimulate plant growth and response to water stress. CO_2_ enrichment could also cause signal transmission, depending on this evidence. Advance studies show that CO_2_ enrichment-induced water stress responses in plants can simulate several signals. These signals are significant in describing the regulatory and molecular mechanisms of CO_2_ enrichment and in accelerating the potential CO_2_ enrichment application in enhancing water stress tolerance in cucumber plants. At the end, enrichment soil with CO_2_ improve the growth performance, photosynthesis rates, production and fruit quality as well as alleviating the water deficit by regulating aquaporin-relative genes.

## Conclusions

This study investigated the combined impact of drought and elevated soil CO_2_ on the yield and physiological responses of cucumber plants, revealing intricate interactions and significant outcomes. Under drought stress conditions, as anticipated, there was a notable decrease in yield and its components compared to well-watered conditions. Interestingly, the 700 ppm CO_2_ treatment emerged as the most effective in enhancing fruit weight under drought stress, highlighting its potential in mitigating the negative effects of water scarcity on cucumber plants. The number of fruits per plant, total yield, and early yield exhibited significant increases under both CO_2_ concentrations in both water conditions. Water productivity, however, demonstrated a direct relationship with CO_2_ concentration until 700 ppm, after which a decline was observed at 1000 ppm. Remarkably, water productivity under 75% ETc with 700 ppm CO_2_ rivaled that of 100% ETc with 1000 ppm CO_2_, suggesting a potential avenue for water conservation without compromising yield significantly. Physiological parameters such as photosynthetic rate, transpiration rate, stomatal conductance, and CO_2_ concentration in stomata were influenced by both water stress and CO_2_ concentrations. Notably, 700 ppm CO_2_ proved to be the most effective in enhancing photosynthetic rate, even surpassing the control under well-watered conditions. Plant height, leaf number, SPAD reading, and biomass all exhibited responses to varying degrees under different treatments, emphasizing the intricate interplay between water availability and CO_2_ concentrations. Furthermore, the study delved into the molecular mechanisms, revealing the regulation of aquaporin-related and stress-responsive genes under water deficit stress and CO_2_ enrichment. The upregulation of aquaporin-related genes and stress-responsive Argonaut (CsSAGO1) genes in response to 700 ppm CO_2_ treatment suggested a potential role in improving water stress tolerance in cucumber plants. The current investigation concluded with a number of rewards, one of them being enhanced commercial production while preserving water—an aim that greenhouse cucumber growers mainly target. In addition, further experiments should be performed to understand well the impacts of systematic elevation of soil carbon dioxide on root architecture and soil microbial activity.

## Data Availability

The raw data will be available on request. Correspondence and requests for materials should be addressed to H.S.O.
